# Distinct mutations in importin-β family nucleocytoplasmic transport receptors transportin-SR and importin-13 affect specific cargo binding

**DOI:** 10.1038/s41598-021-94948-1

**Published:** 2021-08-02

**Authors:** Makoto Kimura, Kenichiro Imai, Yuriko Morinaka, Yoshiko Hosono-Sakuma, Paul Horton, Naoko Imamoto

**Affiliations:** 1 Cellular Dynamics Laboratory, RIKEN Cluster for Pioneering Research, Wako, Saitama Japan; 2 Cellular and Molecular Biotechnology Research Institute, National Institute of Advanced Industrial Science and Technology (AIST), Tokyo, Japan; 3 Molecular Profiling Research Center for Drug Discovery, National Institute of Advanced Industrial Science and Technology (AIST), Tokyo, Japan; 4 Department of Computer Science and Information Engineering, National Cheng Kung University, Tainan City, Taiwan

**Keywords:** Protein transport, Protein-protein interaction networks

## Abstract

Importin-(Imp)β family nucleocytoplasmic transport receptors (NTRs) are supposed to bind to their cargoes through interaction between a confined interface on an NTR and a nuclear localization or export signal (NLS/NES) on a cargo. Although consensus NLS/NES sequence motifs have been defined for cargoes of some NTRs, many experimentally identified cargoes of those NTRs lack those motifs, and consensus NLSs/NESs have been reported for only a few NTRs. Crystal structures of NTR–cargo complexes have exemplified 3D structure-dependent binding of cargoes lacking a consensus NLS/NES to different sites on an NTR. Since only a limited number of NTR–cargo interactions have been studied, whether most cargoes lacking a consensus NLS/NES bind to the same confined interface or to various sites on an NTR is still unclear. Addressing this issue, we generated four mutants of transportin-(Trn)SR, of which many cargoes lack a consensus NLS, and eight mutants of Imp13, where no consensus NLS has been defined, and we analyzed their binding to as many as 40 cargo candidates that we previously identified by a nuclear import reaction-based method. The cargoes bind differently to the NTR mutants, suggesting that positions on an NTR contribute differently to the binding of respective cargoes.

## Introduction

Human cells have approximately 20 species of Impβ family NTRs, which share the task of transporting approximately 35% of expressed proteins. Each of the NTRs has been reported to transport a specific group of cargo proteins or RNAs into or out of the nuclei through the nuclear pores^1,2^, and recent comprehensive analyses further indicated that predominant protein groups in the cargo cohorts are unique to the respective NTRs^3,4^. Thus, the specific NTR–cargo interaction is expected to be attributed to contact between a confined site on the NTR and an NLS/NES on the cargo. Several NTR-specific NLSs/NESs have been described as short regions with consensus sequences. The most well-studied NLS is the classical NLS^5,6^ that binds to Impα, which is a cargo-binding adaptor exclusively for Impβ^7–11^. Sequences similar to the Impβ binding (IBB)-domain in Impα act as NLSs that bind directly to Impβ^12–14^. Other known NLSs/NESs that bind directly to Impβ family NTRs are the PY-NLS for Trn1 and Trn2^15–17^, the Leu-rich NES for CRM1^18–20^, the SR-domain for TrnSR^21^, the IK-NLS for yeast Kap121p^22,23^, and the indefinite β-like importin binding (BIB)-domain, which binds to several NTRs^24^. In addition, the RG/RGG-rich segment for Trn1 and the RSY-rich segment for TrnSR were reported recently^25^. Crystallographic studies revealed the binding structures of these NLSs/NESs and the Impβ family NTRs: IBB-domain/Impβ^26–28^, PY-NLS/Trn1^15,29–33^, NES/CRM1^34–39^, SR-domain/TrnSR^40,41^, and IK-NLS/Kap121p^22,23,42^. In these structures, the NTRs interact with the NLSs/NESs by a confined interface located on a distinct part of each NTR. Some of these structures include only a short peptide containing the NLS/NES, but other structures involve wider cargo domains in addition to the NLS/NES, and in such structures, cargo regions apart from the canonical NLS/NES also interact with the NTR^34,39,41^.

Even if a consensus NLS/NES sequence for an NTR is evident, many cargoes of the same NTR may not have the consensus sequence, and notably, for many NTRs, no consensus NLS/NES sequences have been reported. Recent extensive cargo or binding partner identifications failed to uncover novel consensus NLS/NES sequences^3,4,43–46^. Crystal structures of NTRs complexed with cargoes lacking the canonical NLS/NES have also been determined: Impβ^47–49^, Imp9^50^, Imp13^51–53^, Kap122p^54^, CSE1^55^, exportin-(Xpo)4^56^, Xpo5^57^, and Xpot^58^. In these structures, the NTRs interact with not just a short sequence of the cargo, but rather with multiple sites or a surface arranged conformationally, and respective cargoes bind to different positions on the NTRs. Impβ import cargoes, Snail, PTHrP, and SREBP-2 contact Impβ at multiple distinctive sites, which also differ from the IBB-interface^47–49^. Imp13 alternatively binds to import cargoes, UBE2I (UBC9) and MAGOH (Mago), by its N- and C-terminal region, respectively^51,52^, and an export cargo, EIF1A, by a middle region adjacent to the MAGOH-binding region^53^. In the complex of the Impβ/Imp7 heterodimer and histone H1, fuzzy and nonspecific interactions by disordered regions of the cargo also support complex formation^59^. Thus, it can be postulated that NTRs recognize cargoes lacking a canonical NLS/NES using different contact sites, as required by various three-dimensional cargo structures. Likewise, cargoes containing an NLS/NES may also contact varying NTR sites by regions other than the NLS/NES. Therefore, to understand the specificity determinant of the NTR–cargo interaction, the binding of NTRs to a number of varying proteins or domains lacking a canonical NLS/NES must be characterized.

A substantial number of cargoes or binding partners of NTRs have been identified in recent large-scale studies^3,4,43–46^, but we cannot expect immediate structure determination of so many NTR–cargo complexes. Biochemical protein–protein binding assays using mutant proteins can infer amino acid residues involved in the binding. If mutations located at distinct positions of an NTR affect the binding of different cargoes, that suggests that the cargoes bind to the NTR in different configurations.

Based on extrapolation from reported crystal structures, we hypothesized that cargoes lacking a consensus NLS/NES bind differently to the same NTR. To test this hypothesis, we generated a series of mutants of two human NTRs, TrnSR, of which many identified cargoes lack the SR-domain, which is regarded as its canonical NLS, and Imp13, for which no consensus NLS has been defined, and analyzed the binding to the previously identified cargoes by bead halo assay (BHA). For that purpose, we used NTR mutants already reported to be deleterious to cargo binding and made new mutants, considering the crystal structures and functional differentiation^60,61^, as TrnSR and Imp13 are one of the close paralogous pairs within the Impβ family. To examine TrnSR and Imp13 cargoes, we prepared approximately 40 candidate cargoes each that we identified previously by SILAC-Tp—a combination of stable isotope labeling by amino acid in cell culture (SILAC), an in vitro transport system that imports proteins from nuclear extract into the nuclei of permeabilized cells, and LC–MS/MS^3^. The cargoes showed widely varying spectra of mutant NTR binding, suggesting that the configurations of NTR–cargo interactions are more widely diversified than expected.

## Results

### TrnSR, Imp13, and their cargoes

Previously, we identified candidate cargoes of the 12 import NTRs by the SILAC-Tp method^3^. In those experiments, the plasma membranes of cells labeled with stable isotopes were permeabilized, and unlabeled proteins in a nuclear extract were imported into the nuclei of the permeabilized cells by an NTR of interest. After a pair of import reactions with (+) and without (−) the NTR, the nuclear proteins were extracted and analyzed by LC–MS/MS to derive the unlabeled/labeled (imported/endogenous) quantitation ratio of each protein. From these data, the proteins were ranked in order of the Z-score (standard deviation value) of the log [(unlabeled/labeled)_+NTR_/(unlabeled/labeled)_-NTR_] ratio. The experiments were triplicated for each NTR. Proteins ranked in the top 4% (49–66 proteins) by the lowest (3rd) Z-score in triplicate (3rd-Z-4% cargoes) were assessed as reliable candidate cargoes, whereas the top 15% (245–309 proteins) by the 2nd Z-score were assessed as possible candidate cargoes (2nd-Z-15% cargoes). Contrary to expectation, we were unable to discern any novel consensus sequence within the identified cargoes, raising the question of how these proteins bind to the NTRs. Thus, we analyzed the binding of the identified cargoes. We focused on TrnSR and Imp13 because (1) some cargo-binding crystal structures are available, (2) many of the identified TrnSR cargoes lack the canonical NLS, SR-domain, and no consensus NLS has been defined for Imp13 cargoes, suggesting that these NTRs provide interfaces structurally distinct from those for canonical NLS interactions, and (3) TrnSR and Imp13 are one of the closely homologous pairs in the Impβ family, but they share few cargoes^3^ and therefore are a convenient pair for the prediction of specific cargo-binding sites acquired after evolutionary divergence (see the next section). For cargo specimens, we employed 3rd-Z-4% cargoes. Of the 65 and 66 TrnSR and Imp13 3rd-Z-4% cargoes, 41 and 40 cargoes, respectively, were successfully prepared as a GFP-fusion protein in a bacterial extract and quantified by triplicate Western blotting with an anti-GFP antibody (Supplementary Fig. S1). Here, we refer to the cargoes by their gene names and 3rd-Z-rank order in our previous paper^3^.

### Selection of sites to mutate in TrnSR and Imp13

**Figure 1:**
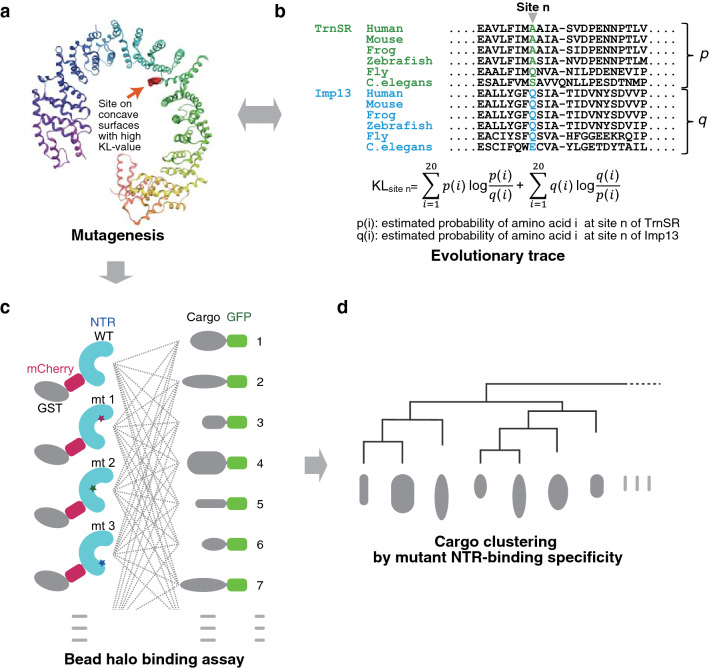
Research workflow. (**a**) In addition to amino acid residues that contact cargoes in reported crystal structures, residues exposed on the concave surface facing the cavity of the TrnSR or Imp13 structure were considered candidate sites for mutational analysis. (**b**) Residues conserved across species within either TrnSR or Imp13 orthologs but not between them were identified via an ETA of 78 and 73 metazoan TrnSR and Imp13 sequences, respectively. The sequences were aligned, and the obtained amino acid frequencies of each column were used to estimate probabilities for use in computing the symmetric Kullback–Leibler divergence (KL value) for each aligned position (site n). (**c**) WT and mutagenized (mt) TrnSR and Imp13 (NTR) and their cargoes were prepared as bacterially expressed GST-mCherry-NTR fusion and GFP-cargo fusion proteins, respectively. Binding between the GST-mCherry-NTRs and the GFP-cargo was analyzed in all combinations by BHA. GST-mCherry-NTR was fixed on GSH-Sepharose beads and mixed with a bacterial extract containing the GFP-cargo protein. The beads were then observed with a confocal microscope, and the binding intensity was quantified from the fluorescence of mCherry and GFP in the images. (**d**) The cargoes were clustered based on their NTR mutant-binding specificity.

To analyze the effects of NTR mutations on the binding of various cargoes, we needed to design NTR mutants that are highly likely to be defective in binding to some cargoes. We expected that analyses focusing on a couple of distinct regions on the NTRs would yield sufficient information to illustrate the similarity or variety of the binding configurations and considered that the effects of mutations in close proximity should also be compared by introducing a few mutations into a small region. Thus, we selected the residues to substitute in such a way as to form a couple of separated clusters on the NTR; with priority order: (1) residues in contact with cargoes in crystal structures and whose mutations were defective in cargo binding in pull-down assay, (2) residues in contact with cargoes but not assayed by pull-down, and (3) residues facing towards cargoes in crystal structures and predicted as evolutionarily differentiated cargo-binding sites by high symmetric Kullback–Leibler information values (KL values) in a modified evolutionary trace analysis^60^ (ETA, see the next paragraph) (Fig. 1a, b). The selected regions differed between TrnSR and Imp13 because more cargo-contact sites were found, and more mutants were reported for Imp13 than TrnSR. We substituted charged residues with oppositely charged residues because residues were replaced similarly in previously characterized mutants defective in cargo binding^41,51,52^ (*e.g.*, TrnSR-R671E, -D750R/D751R and Imp13-K802E/R803E).

ETA aims to correlate sequence variations with differentiated protein functions^60,61^. Impβ family NTRs share primary structures, although the amino acid identities between quite a few NTRs are less than 30%, and any pair within the 12 import NTRs shares a certain number of the identified cargoes. In general, homologous NTRs share many cargoes^3^. However, TrnSR and Imp13, which are so similar as to form one of the closest pairs in the phylogenetic analysis of 12 Impβ family import NTRs, are functionally differentiated to share few cargoes^3^. This is a distinctive feature of this pair, and we expected that cargo-binding sites evolutionarily differentiated between TrnSR and Imp13 could be predicted by ETA. ETA focuses on amino acid positions conserved in each paralog but not across the paralogs, *e.g.*, positions consistently occupied by one amino acid in TrnSR sequences and another amino acid in Imp13 sequences. Such positions are presumably responsible for the functional differentiation between the paralogs in general^60,61^ and, in particular, the differential cargo recognition of TrnSR and Imp13. TrnSR and Imp13 are close homologs but do differ consistently in a few positions. To systematically identify such positions, we performed a modified ETA using a symmetric KL value^60^ (Fig. 1b and see Methods). The KL value and the estimated amino acid probability of each position of human TrnSR and Imp13 are presented in Supplementary Table S1. We considered positions with a high (within the top 10%) KL value as the candidate sites for mutagenesis.

**Figure 2:**
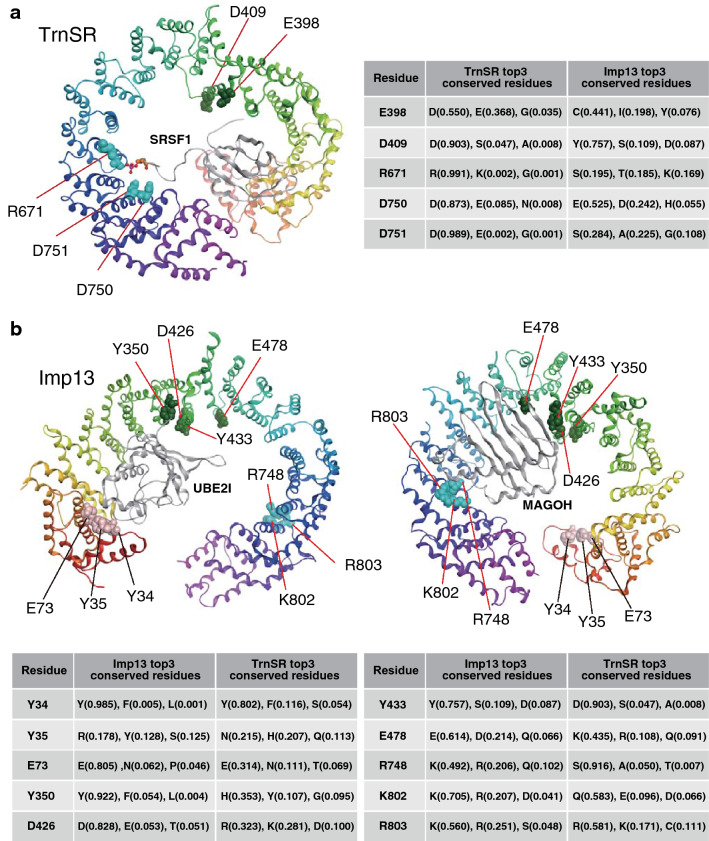
Substituted amino acid residues. (**a**) Positions of the five residues substituted in TrnSR are indicated on the structure of TrnSR binding to SRSF1 (ASF/SF2, gray). The three most conserved amino acids in those positions in the 78 metazoan TrnSR orthologs are shown on the right, with probabilities estimated from their amino acid frequencies. The residues were substituted with oppositely charged amino acids. The D750/D751 dipeptide was substituted in one construct following a previous work^41^. E398 and D409 are proximal, and R671 and D750/D751 are also proximal. See also Table 1. (**b**) The ten residues substituted in Imp13 are indicated on the structures of Imp13 binding to UBE2I (UBC9, gray) (left) or MAGOH (Mago, gray) (right). The three most conserved amino acids in those positions in the 73 metazoan Imp13 orthologs are shown with probabilities estimated from their amino acid frequencies. These residues were also substituted with oppositely charged amino acids. Two constructs have dipeptide substitutions at Y34/Y35 and K802/R803 following a previous work^52^. These residues reside in three separated regions: N-terminal region, Y34/Y35 and E73; middle, Y350, D426, Y433, and E478; and C-terminal, R748 and K802/R803. See also Table 1.

According to these considerations, we generated four TrnSR substitution mutants: TrnSR-E398R, -D409R, -R671E, and -D750R/D751R double mutant (Fig. 2a and Table 1). R671 and D750/D751 are located near each other in the crystal structure, and interact with the SR-domains of cargoes SRSF1 (ASF/SF2) and CPSF6^40,41^. TrnSR-R671E and -D750R/D751R were characterized by pull-down assay^41^. As expected, the alignment sites of R671 and D751 have high KL values (Table 1 and Supplementary Table S1). We selected E398 and D409, located near each other, because they are exposed toward a cargo, SRSF1, and their alignment sites have high KL values. The small region in the TrnSR structure that includes E398 and D409 and another region that includes R671 and D750/D751 are opposed to each other across the cavity (Fig. 2a).

**Table 1:** TrnSR and Imp13 mutants used.

NTR	Amino acid substitution	KL value (top 10% > 7.5)	Cargo binding analyzed by pull-down assay	Cargo contact in crystal structure
TrnSR	E398R	13.966		
	D409R	10.496		
	R671E	8.310	SRSF1, CPSF6^41^	SRSF1^41^, CPSF6^40^
	D750R/D751R	2.980/13.051	SRSF1, CPSF6^41^	SRSF1^41^, CPSF6^40^
Imp13	Y34R/Y35R	0.957/3.615	UBE2I^52^	UBE2I^52^
	E73R	2.626		UBE2I^52^
	Y350R	7.590		
	D426R	6.809	UBE2I^52^	UBE2I^52^
	Y433R	10.496	*Drosophila* Mago-Y14^51^ (*Drosophila* Imp13-Y447)	*Drosophila* Mago^51^ (*Drosophila* Imp13-Y447)
	E478R	7.014		*Drosophila* Mago^51^ (*Drosophila* Imp13-E493)
	R748E	12.836		
	K802E/R803E	8.877/2.293	MAGOH-RBM8A (Mago-Y14)^52^ *Drosophila* Mago-Y14^51^ (*Drosophila* Imp13-K814/K815)	*Drosophila* Mago^51^ (*Drosophila* Imp13-K814/K815)

We also generated eight Imp13 mutants: Imp13-Y34R/Y35R, -E73R, -Y350R, -D426R, -Y433R, -E478R, -R748E, and -K802E/R803E (Fig. 2b and Table 1). Y433, E478, and K802/R803 correspond to Y447, E493, and K814/K815, respectively, in *Drosophila* Imp13. Y34/Y35 and E73 are located near each other in the crystal structure. Imp13-Y34R/Y35R was characterized by pull-down assay, and Y34/Y35 and E73 interact with a cargo, UBE2I, in the structure^52^. However, their KL values were unexpectedly low (Table 1 and Supplementary Table S1). Imp13-D426R and -Y433R were characterized by pull-down assay^51,52^. D426 contacts UBE2I^52^, and Y433 and E478 contact a cargo, MAGOH^51^. Y433 has a high KL value, while the KL values of D426 and E478 are somewhat lower than the top 10% KL value. However, in the D426 and E478 positions, residues charged oppositely between TrnSR and Imp13 are conserved through species (Fig. 2b). Y350 was selected in consideration of its structural position and high KL value. Y350, D426, Y433, and E478 locate nearby one another. Imp13-K802E/R803E was characterized by pull-down assay, and K802/R803 interacts with MAGOH^51^. R748 was selected because of its structural position near K802/R803 and its high KL value. These residues form three clusters on the concave surface of Imp13: N-terminal cluster, Y34/Y35 and E73; middle, Y350, D426, Y433, and E478; and C-terminal, R748 and K802/R803 (Fig. 2b).

### Bead halo assay

**Figure 3:**
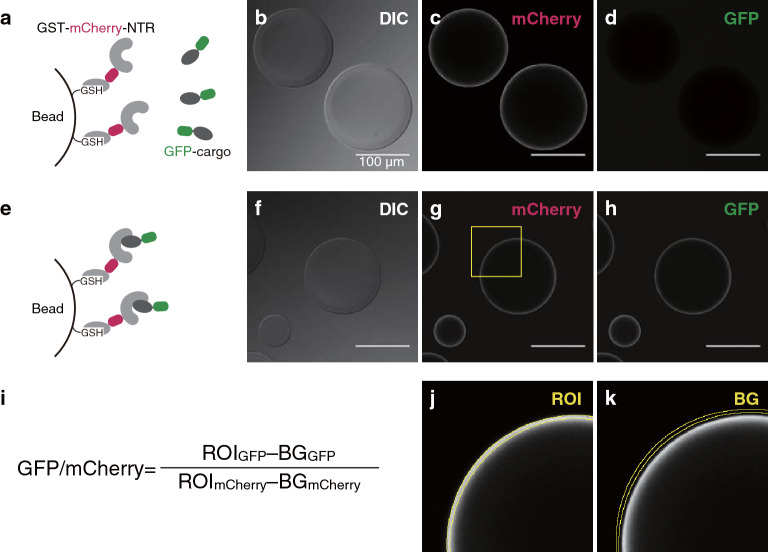
Bead halo assay. (**a–d**) An example of an NTR–cargo pair that does not bind. **(e–h)** An example of an NTR–cargo pair that does bind. **(a)** A GST-mCherry-NTR is fixed on GSH-Sepharose beads. If GFP-cargo does not bind to the NTR, it remains in the buffer around the beads. **(e)** If cargo binds to the NTR, it is concentrated on the bead surface. **(b,** **f)** Differential interference contrast (DIC) microscopic images of the beads. **(c, g)** Confocal microscopic images of mCherry fluorescence. The yellow square in (**g**) indicates the magnified region in (**j**, **k**). **(d, h)** Confocal images of GFP fluorescence. **(i)** After background subtraction, the GFP/mCherry fluorescence ratio around the beads was used as the index for the binding intensity. ROI and BG were defined as in (**j**, **k**). The region mean intensities in 12-bit images were used for the calculation. **(j)** The ROI was set on the mCherry fluorescent image as ring-shaped regions with a 5 pixel width along the inside of the outlines of beads (yellow double line). The same ROI was applied to the corresponding GFP fluorescent image. **(k)** The BG was established as larger ring-shaped regions with a 5 pixel width and 5 pixels away outside the ROI. Images in (**j, k**) are magnifications of the square region in (**g**). In the actual quantitation, ROIs and BGs were set on original images with dimensions of 1024 × 1024 pixels, of which the field size (317 × 317 µm) was the same as that of the images (b–d and f–h). Scale bar: 100 µm. For more examples of the ROI setting, see Supplementary Fig. S2.

We analyzed the binding of the 3rd-Z-4% cargoes to TrnSR and Imp13 by BHA^62^ (Figs. 1c, 3). The TrnSR and Imp13 proteins were expressed in bacteria and purified as GST-mCherry-TrnSR and GST-mCherry-Imp13 red fluorescent fusion proteins, respectively. The GST-mCherry-TrnSR or -Imp13 protein was fixed on glutathione (GSH)-Sepharose beads and mixed with an extract containing one of the GFP-cargo fusion proteins. The beads were observed with a confocal laser scanning microscope, and the fluorescent intensities of mCherry and GFP along the rims of the beads were measured from the confocal images (Fig. 3). In the images, only beads around which regions of interest (ROIs) and background (BG) regions can be set as unbroken circles were selected for measurement (Supplementary Fig. S2), yielding one or two beads as the ROIs for most images. We employed the BG-subtracted GFP/mCherry fluorescent ratio as the index for the binding.

**Figure 4:**
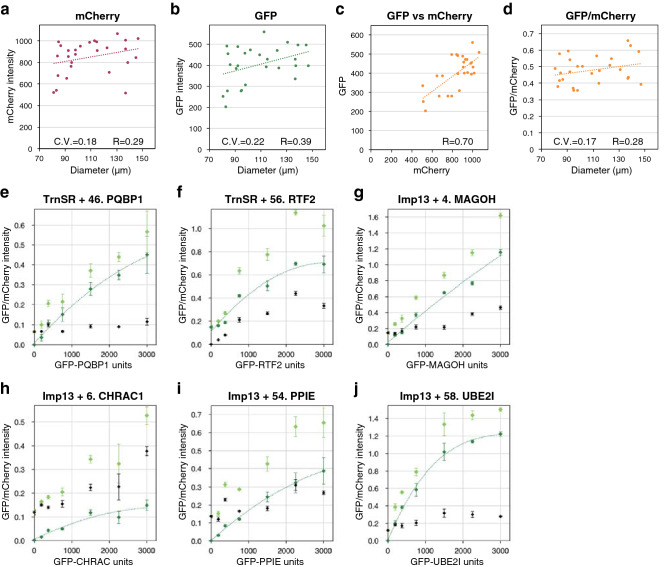
Evaluation of the bead halo assay system. **(a–d)** The fluorescence intensity of GST-mCherry-TrnSR and GFP-SRSF1 as measured for 27 single beads is shown to evaluate the deviation of assay values. **(a)** BG-subtracted mCherry, **(b)** BG-subtracted GFP, and **(d)** BG-subtracted GFP/mCherry values are plotted against the bead diameters. **(c)** BG-subtracted GFP intensities are plotted against the BG-subtracted mCherry intensities of the same beads. C.V., coefficient of variation of the fluorescent values; R, correlation coefficient between the fluorescent values and the bead diameters (**a, b, d**) or between the mCherry and GFP intensities (**c**). **(e–j)** The dosage of bacterial extract containing GFP-cargo was varied from zero to that used in the final assay to evaluate the dose–response. **(e)** GST-mCherry-TrnSR and GFP-PQBP1, **(f)** GST-mCherry-TrnSR and GFP-RTF2, **(g)** GST-mCherry-Imp13 and GFP-MAGOH, **(h)** GST-mCherry-Imp13 and GFP-CHRAC1, **(i)** GST-mCherry-Imp13 and GFP-PPIE, and **(j)** GST-mCherry-Imp13 and GFP-UBE2I were analyzed by BHA. Numbers are the 3rd-Z-ranks of the cargoes. Three images for one GFP-cargo dosage were quantified, and the mean intensities of the GFP without BG subtraction (light green), BG of GFP (black), and BG-subtracted GFP (green) divided by the mean BG-subtracted mCherry intensity are plotted with the S.D. indicated by the error bar. Note that the BG of mCherry is low because the beads were used after washing out the unbound GST-mCherry-NTR, while the BG of GFP rises with the dose of GFP-cargo because it includes the fluorescence of free GFP-cargo that is in equilibrium with bead-bound GFP-cargo. All the NTR used are WT. The fitted curves were drawn by Microsoft Excel.

We fused mCherry into GST-NTR proteins and used the GFP/mCherry fluorescent ratios as the index to improve quantitation accuracy. Initially, we assessed the deviation of the measured values using GST-mCherry-TrnSR and GFP-SRSF1 as an example (Fig. 4a–d) to evaluate our experimental system. In all BHAs, we selected beads 80–150 µm in diameter for quantitation. In this range, intensities of neither mCherry nor GFP fluorescence correlated with the diameter, although both fluctuated independently of the diameter (Fig. 4a, b). However, mCherry and GFP intensities correlated with each other (R = 0.70, Fig. 4c), representing that GFP-cargo binds to a bead in proportion to the amount of GST-mCherry-NTR on the bead. As seen in some images in this report (*e.g.*, Fig. 3), some ROIs contain uneven fluorescence, but in most cases, mCherry and GFP show similar uneven fluorescence patterns, indicating proportional binding of GFP-cargo to GST-mCherry-NTR at any place on a bead. Accordingly, the coefficient of variation (C.V. = S.D./mean), which was 0.22 for GFP (Fig. 4b), was reduced to 0.17 for GFP/mCherry (Fig. 4d). All the GFP/mCherry values in Fig. 4d are within the mean ± 36% range, and a significant reduction (*e.g.*, > 40% reduction) in GFP/mCherry values in the following assays of NTR mutants assuredly indicates reduced binding, albeit not precisely quantitative. Note that in the final assays (Supplementary Table S2a and b), the GFP/mCherry ratios of three images were averaged and used to calculate the normalized GFP/mCherry values, and therefore, the measurement errors were reduced. In the negative control assay, intact GFP bound to TrnSR weakly, though not to Imp13 (Supplementary Fig. S3a and e). We applied different thresholds for negative binding to TrnSR (GFP/mCherry without normalization, 0.1) and Imp13 (0.05) to avoid unreliable quantification at a low range (Supplementary Fig. S3b–d).

Next, we assessed the dose–response of the system using two TrnSR- and four Imp13-specific cargoes (Fig. 4e–j). The amount of extract containing GFP-cargo was varied from zero up to that used in the final assays of NTR mutants. In some cases, the BG-subtracted GFP/mCherry ratio responded to the GFP-cargo dose almost linearly (Fig. 4e, g, i), and reduced binding to NTR mutants would be detected sensitively. In other cases, the BG-subtracted GFP/mCherry ratio approached saturation (Fig. 4f, h, j), and a slight reduction in binding would be challenging to detect. Thus, our assays should reliably detect reductions in binding affinity but perhaps with imperfect sensitivity in some cases. In the final assays of NTR mutants below, GFP-cargoes of equal amount were used when possible (see the legend of Supplementary Table S2).

Then, we checked the reproducibility of the quantitation (Supplementary Fig. S4a–f left panels), with particular attention to Imp13 since the Imp13 BHA results were more varied and involved more mutants than those of TrnSR. The binding of two TrnSR- and four Imp13-specific cargoes to the WT and mutant NTRs were analyzed three times. The three mean GFP/mCherry values of most triplicates agreed reasonably well with one another. Although the GFP-cargoes used in this reproducibility analysis were prepared independently of those used in the final assays (see Methods), the GFP/mCherry values of mutant NTRs normalized to that of WT were roughly consistent with those in the final assay (Supplementary Fig. S4a–f right panels). Notably, a significant reduction in cargo binding of an NTR mutant could consistently be observed in the repeated experiments.

We compared the binding of WT and mutant NTRs to the same cargo by localized surface plasmon resonance (LSPR) to estimate the physical significance of the major binding reduction observed for NTR mutants (Supplementary Fig. S5). Further, we prepared NTR and cargo proteins without GFP- and mCherry-fusion to avoid optical interference. A His_6_-tagged cargo protein was fixed on a Ni^2+^-charged nitrilotriacetic acid (NTA) sensor chip, and the kinetic association and dissociation of a GST-NTR on the sensor were analyzed. Although we tried several NTR–cargo combinations, we could only set up adequate experimental conditions for the TrnSR–DNAJB1 interaction. In our LSPR experiment, TrnSR-WT bound strongly to DNAJB1 with K_D_ ~ 7.4 nM (Supplementary Fig. S5a), comparable to the highest level of NTR–cargo affinity reported for export NTRs in the presence of RanGTP; CRM1–SNUPN (K_D_ ~ 10 nM)^63^, Xpot–tRNA (K_D_ ~ 2 nM)^64^, and Xpo4–hypusinated EIF5A (K_D_ ~ 2 nM)^65^. Strong cargo binding may be an intrinsic feature of TrnSR because TrnSR-WT generally exhibited much higher unnormalized GFP/mCherry ratios than Imp13-WT (Supplementary Table S2a and b). In the LSPR, TrnSR-D750R/D751R bound to DNAJB1 with K_D_ ~ 28 nM (Supplementary Fig. S5b), indicating an ~ 3.8-fold affinity reduction from the WT. In the BHAs, the BG-subtracted GFP/mCherry ratios of TrnSR-D750R/D751R were as low as 3–25% of the WT in the reproducibility analysis and the final assay (Supplementary Fig. S5c and d, left panels), and the reductions in the GFP/mCherry ratios seem to be more than can be expected from the K_D_ shift in LSPR. However, the GFP/mCherry ratios of TrnSR-D750R/D751R without BG subtraction were 14–52% of WT (Supplementary Fig. S5c and d, right panels), which was more consistent with the affinity reduction in the LSPR. We set BG regions outside beads to highlight the difference in the GFP/mCherry ratios of the ROIs, but the BG regions contain free GFP-cargoes yielding higher signals than the ROIs without GFP-cargo binding (see Supplementary Fig. S3b) because free GFP-cargoes do not penetrate beads. Since TrnSR-D750R/D751R showed significantly reduced cargo binding in our BHA, we conclude that our system can detect binding reductions of less than one order of magnitude.

### Binding of the cargoes to the WT NTRs

Imp13 is a bidirectional NTR^66^, but the candidate cargoes are most likely to be import cargoes because they were identified by the import reaction-based method SILAC-Tp (see the section, TrnSR, Imp13, and their cargoes). As described in the previous paper^3^, an export cargo of Imp13, EIF1AX^66^, ranked relatively high (164th in 1671 proteins) by the 3rd-Z-score. The unintended export of endogenous EIF1AX by Imp13 must have raised the imported/endogenous (unlabeled/labeled) ratio in the experiment. Judging by the absolute levels of imported (unlabeled) and endogenous (labeled) proteins in the LC–MS/MS, EIF1AX, abundant in the nuclei, seemed to be the only export cargo ranked high^3^.

NTR–cargo complexes pass through the nuclear pores by diffusion, and the transport direction is regulated at the steps of association and dissociation of the complexes in the nuclei and cytoplasm^67^. RanGTP, which is rich in the nuclei and converted into RanGDP in the cytoplasm, promotes the association and dissociation of export and import NTR–cargo complexes, respectively. We analyzed the binding of all the prepared cargoes in the presence or absence of a GTP-fixed mutant of Ran, Q69L-RanGTP, by BHA (Supplementary Fig. S6). Most of the candidate cargoes that we could analyze bound to their respective NTRs, (35 out of 41 TrnSR cargoes and 27 out of 40 Imp13 cargos), and in most cases except the Imp13 export cargo EIF1AX, the addition of Q69L-RanGTP decreased the GFP/mCherry values (Supplementary Fig. S6, 3rd-Z-rank of EIF1AX is 164 of Imp13), supporting the functionality of the binding. This result attests to the reliability of the SILAC-Tp method, especially when considering that BHAs can detect only the direct binding of a protein to an NTR, but the SILAC-Tp method can also identify indirect cargoes that bind to an NTR by forming complexes with direct cargoes. Piggybacking nuclear import is prevalent in human cells^68^, and in the STRING database^69^, quite a few 3rd-Z-4% cargoes of which direct NTR binding was not detected are expected to interact with other 3rd-Z-4% cargoes that bound directly to the NTR (Supplementary Table S2c). Since many unidentified cargoes may also connect the NTRs and indirect cargoes, the possibility of piggybacking may be high for the NTR-unbound cargoes. However, we should note that binding inhibition in BHA by the GFP-fusion to cargoes is possible. Inhibition of binding by the N-terminal fusion of GST-mCherry to the NTR is improbable, albeit not impossible, because UBE2I and RanGTP interact with the N-terminal region of the NTR^41,51,52^ and GST-mCherry-NTR. In any case, the candidate cargoes that do not bind to the NTRs in BHA could still be authentic.

### Cargoes bind to the TrnSR and Imp13 mutants with varying affinities

**Figure 5:**
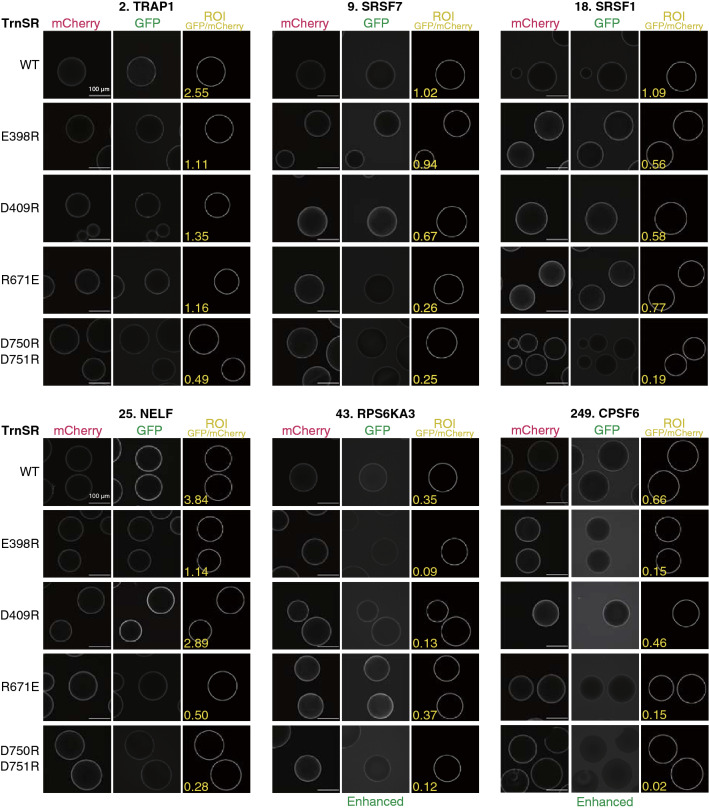
Bead halo assay of TrnSR mutants. Examples of the GFP and mCherry fluorescent images used to quantify the binding of six GFP-cargoes (number: 3rd-Z-rank) to the WT and four mutants of GST-mCherry-TrnSR are shown. ROIs used for the quantitation are indicated by white circles. BG-subtracted GFP/mCherry ratios calculated from these image values are also designated in the ROI images. Note that these GFP/mCherry ratios are not normalized by the WT ratio and that they slightly differ from those in Supplementary Tale S2a, which are the means of three images. The GFP images of RPS6KA3 and CPSF6 are enhanced equivalently. Scale bar: 100 µm.

**Figure 6:**
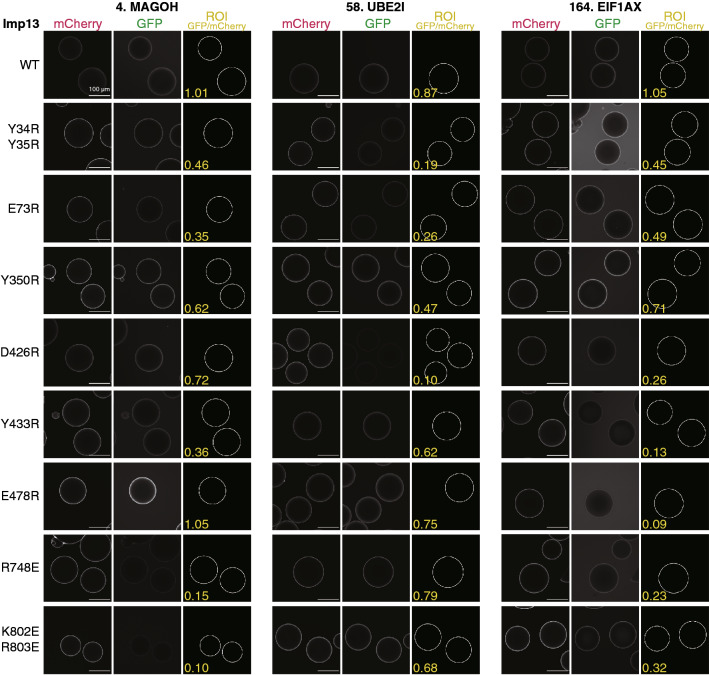
Bead halo assay of Imp13 mutants. Examples of the GFP and mCherry fluorescent images used to quantify the binding of three GFP-cargoes (number: 3rd-Z-rank) to the WT and eight mutants of GST-mCherry-Imp13 are shown. ROIs used for the quantitation are specified by white circles. BG-subtracted GFP/mCherry ratios calculated from these image values are also indicated in the ROI images. Note that the GFP/mCherry ratios are not normalized by the WT ratio and that they slightly differ from those in Supplementary Tale S2b, which are the mean of three images. Scale bar: 100 µm.

We comprehensively analyzed the binding of 3rd-Z-4% cargoes to the TrnSR and Imp13 mutants by BHA. Examples of the images and GFP/mCherry ratios are shown in Figs. 5 and 6. The GFP/mCherry ratios of the NTR mutants were normalized by the WT NTR ratio for the same cargo to evaluate the mutational effects on the binding of respective cargoes (Supplementary Table S2a and b). Considering the estimated accuracy of the BHA (Fig. 4), we discretized the normalized GFP/mCherry values into three ranks: not reduced (> 0.60), reduced (≤ 0.60 and > 0.20), and much reduced (≤ 0.20).

Most of the TrnSR cargoes that bound to TrnSR-WT showed markedly reduced binding to TrnSR-D750R/D751R. D750/D751 was reported to interact with the SR-domains as referred above, but only a few of the TrnSR cargoes have an apparent SR-domain, including the recently proposed RSY-rich segment^25^ (Supplementary Table S2a). Thus, D750/D751 should also be involved in the interaction with cargoes lacking an SR-domain. The other mutants, TrnSR-E396R, -D409R, and -R671E, showed diverse effects depending on the combination of the particular mutation and cargo, mostly reducing binding but in a few cases increasing binding (*e.g.*, PPIL1 binding to TrnSR-D409R). Since respective cargoes are affected by different mutations to varying degrees, the cargoes most likely bind to TrnSR in diverse configurations. Likewise, all of the Imp13 cargoes that bound to Imp13-WT exhibited varying affinities to the eight Imp13 mutants, and thus, the cargoes probably differ in how they bind. Examples in Fig. 6 clearly illustrate that respective cargoes are affected by different mutations. Two mutants that reduced the binding most among the eight mutants were Imp13-R741E and -K802E/R803E for MAGOH binding, -Y34R/Y35R and -D426R for UBE2I binding, and -Y433R and -E478R for EIF1AX binding. The involvement of the C-terminal, N-terminal, and middle regions of Imp13 in the binding of MAGOH, UBE2I, and EIF1AX, respectively, agrees with the crystal structures^51–53^. Although EIF1AX is an export cargo of Imp13, they form a complex without RanGTP in the cytoplasm and, RanGTP enhances binding^66^ (Supplementary Fig. S6). Thus, we believe that BHA reflects cytoplasmic complex formation.

### Effects of the NTR mutations do not relate to structural similarity of the cargoes

**Figure 7:**
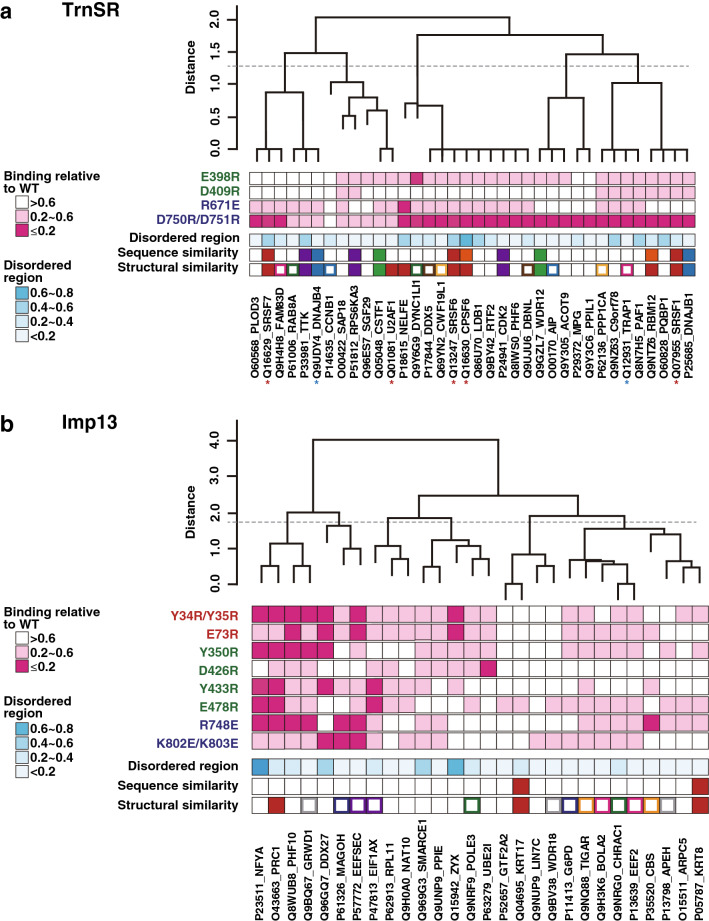
Cargo clustering by the binding specificity to TrnSR or Imp13 mutants. **(a)** The TrnSR cargoes were hierarchically clustered by the binding intensities to the TrnSR mutants. For each cargo, GFP/mCherry ratios of the TrnSR mutants were normalized to WT, and converted into three values, ≤ 0.2 into 0, > 0.2 and ≤ 0.6 into 0.5, and > 0.6 into 1, as indicated by the magenta scale (Supplementary Table S2a). Ward's method was used among the cargoes with the Euclidean distances calculated from these discrete values. The hierarchical clustering was performed using R version 3.5.1. The dashed line on the dendrogram is an arbitrary boundary to separate the cargoes into five groups. The blue scale signifies the content rates of disordered regions, where ten or more consecutive residues are predicted to be disordered. Sequence similarity was analyzed by SSEARCH, and homologous proteins (E-value < 10^–9^) are indicated by the same color. 3D-structural similarity was evaluated by the TM-score using determined or homology modeled structures (Supplementary Table S3a). Proteins with high structural similarity (TM-score > 0.6) are designated by the same solid colors, and those with similarity (0.6 > TM-score > 0.5) are represented by the same frame colors. Red asterisks indicate proteins annotated to have an SR-domain (Arg/Ser-rich domain) in UniProt (https://www.uniprot.org), and blue asterisks indicate an RSY-rich segment^25^ other than the SR-domain. The proximal mutants of TrnSR are specified by the same color. **(b)** The Imp13 cargoes were clustered by the binding intensities to the Imp13 mutants. Illustrated similarly to (a). The dashed line arbitrarily separates the cargoes into six groups.

The cargoes may be classified according to the mutant NTR-binding profiles of the cargoes. We clustered the cargoes using Ward's method to explore this possibility (Figs. 1d and 7). We assigned discrete values to the three ranks of the normalized GFP/mCherry values described in the previous section (Supplementary Table S2a and b) as follows: 1 for > 0.6 (not reduced), 0.5 for ≤ 0.60 and > 0.20 (reduced), and 0 for ≤ 0.20 (much reduced), and used the Euclidean distances of these values as the metric. Both the TrnSR and Imp13 cargoes can be classified into a reasonable number of groups. For example, they can be divided into five (Fig. 7a) or six (b) clusters by setting an arbitrary breakpoint. However, the clusters are not separated discretely; the mutant NTR-binding profiles change gradually through the clusters.

We examined the sequence and structural similarities of the cargoes to relate the mutant NTR-binding specificities and the primary and tertiary structures of the cargoes. From both determined structures and homology modeled structures (Supplementary Table S3a), we calculated structural similarities using MICAN and template modeling scores (TM-scores) of MICAN^70^ for the structure assigned regions of all possible pairs of cargoes. MICAN is a structural alignment algorithm for identifying the best structural alignment between a protein pair by disregarding the connectivity between secondary structure elements, and thus, it can find similar secondary structure packing arrangements with different topologies. Some TrnSR cargoes share a structural similarity, but they are separated in cargo clustering by mutant TrnSR-binding intensities (Fig. 7a); for example, SRSF7, SRSF6, CPSF6, SRSF1, NELFE, and RBM12 have similar RNA recognition motif domains, but these proteins are distributed in the dendrogram based on mutant-binding specificity. Fewer Imp13 cargoes share structural similarity, and they have also clustered apart (Fig. 7b); high structural similarity is detected only among α-helical substructures of KRT8, KRT17, and PRC1. The sequence similarity search results (Fig. 7) and Pfam^71^ domain annotations (Supplementary Table S3a) overlapped with those of the structural similarity search. The fraction of predicted disordered residues of the cargoes also appears irrelevant to the clustering (Fig. 7). Thus, the specificity determination of the NTR–cargo interaction must result from more complicated or fortuitous mechanisms than we expected.

## Discussion

In this work, we analyzed the effects of NTR mutations on cargo binding. Biochemical studies with mutant proteins may involve the risk of distant effects of overall structural collapse. However, most binding reductions observed here are plausibly due to local effects of the mutations because, for each mutation, a certain number of cargoes are unaffected, and the unaffected cargoes differ between mutations, even those which are proximal to each other (Fig. 7 and Supplementary Table S2). Furthermore, in the verifiable cases, the mutant NTR-binding cargo profiles agree with the reported structures (Fig. 6). In our mutagenesis, we preferentially selected residues that contact cargoes in crystal structures regardless of their KL values, and then selected the other residues located near cargoes and exhibiting high KL values, *i.e.*, E398 and D409 of TrnSR and Y350 and R748 of Imp13. As the four mutants are defective in binding to some cargoes, the combination of structural and evolutionary considerations seems useful to predict amino acid positions significant for ortholog function. However, ETA by itself is not sufficient to identify all cargo binding-related residues, because mutations at positions with lower KL values (*e.g.*, Y34/Y35 and E73 of Imp13) also affected cargo binding. Further analysis of more mutants should help further delineate the utility and limitations of ETA in this setting. To analyze NTR–cargo binding, we applied BHA, which is advantageous to analyze many samples but less quantitative than other methods such as isothermal titration calorimetry or SPR. We reduced measurement deviation using the red fluorescent GST-mCherry-NTR proteins and utilizing the GFP/mCherry ratios (Fig. 4). Based on this deviation, we set the threshold for a significant reduction in NTR mutant assays as > 40% reduction from the WT value. Adjusting the concentrations of all the cargoes within their linear ranges of NTR binding is difficult, and some of the cargoes were assayed at near saturation levels (Fig. 4f, h, j). In those cases, a significant reduction in the GFP/mCherry value sufficiently demonstrates reduced affinity to the NTR mutants, but values equal to that of WT do not necessarily indicate a WT level of binding affinity. In the final assays, at least one NTR mutant reduced the GFP/mCherry value for most of the respective cargoes (Fig. 7 and Supplementary Table S2a and b), indicating that the assay condition was sufficient to specify the mutation that most impacted the binding to each cargo. Since the reduced-binding NTR mutants and the degree of the binding reduction differed among the cargoes (Fig. 7 and Supplementary Table S2a and b), respective cargoes are affected differently by a set of NTR mutations, presumably reflecting NTR–cargo binding configuration diversity.

Another potential concern about our BHA is that we used bacterially expressed proteins devoid of eukaryotic posttranslational modifications (PTMs). Many PTMs on both cargoes and NTRs affect binding specificity^2^; for example, hypusinated EIF5A purified from HeLa cells binds to Xpo4–RanGTP complexes ~ 35 times more strongly than unmodified recombinant EIF5A^65^. Thus, our assay may not precisely mirror the binding specificity in vivo, but our results should still reflect the NTR–cargo binding configuration diversity. Degradation products of GFP-cargo proteins in some bacterial extracts used (Supplementary Fig. S1) should also be noted. If degraded cargo fragments have altered affinities to the NTR, the quantitation values in BHA may be deflected. However, the value order of the NTR mutants in binding to a particular cargo can be assumed not to change, because this kind of fragments can usually be regarded to affect uniformly on the values.

Most TrnSR cargoes have neither an SR-domain nor an RSY-rich segment expected to act as an NLS, and no Imp13-specific NLS is known. Therefore, our results suggest that cargoes without a canonical NLS must interact with varying contact sites on an NTR; at least, each position on an NTR contributes to the binding of different cargo sets, and the degree of contribution also differs widely. Thus, the NTRs presumably accommodate the diverse structures of NLS-less cargoes, differentially using multiple contact sites. Although our mutants do not cover all possible cargo contact sites, they sufficiently elucidate the basic mechanism of cargo recognition. Assuming that an NTR uses multiple contact sites in various combinations to interact with structurally diverse cargoes, correlations among the cargo-binding profiles of NTR mutants may reflect the formation of an interface common to some cargoes. The GFP/mCherry values of Imp13-Y34R/Y35R, -E73R, -Y350R, and -Y433R have high correlation coefficients (Supplementary Table S2d), and the N-terminal region containing these sites may form an interface that interacts with the cargoes at partially common and partially distinct contact sites. The binding of UBE2I may be a good example that uses this interface. The cargoes affected by these mutations are clustered proximally in the analysis in Fig. 7b, but we could not find the structural similarity among them. A more advanced type of analysis may be needed to extract protein features useful to predict specific cargoes. Not enough mutants were analyzed from TrnSR or other regions of Imp13 to discuss them in similar detail. Nevertheless, our results support the idea extrapolated from a limited number of crystal structures that NTRs bind to diverse cargoes differently.

TrnSR and Imp13 form one of the closest pairs in the Impβ family phylogram but share fewer cargoes^3^. Although a high KL value in ETA is not essential for a cargo contact site, the selected positions with high KL values appeared significant for the binding of the specific cargoes. Therefore, TrnSR and Imp13 might have diverged evolutionarily from a common ancestral NTR to adapt to bind specific cargoes. In our phylogenetic profiles of TrnSR and Imp13, TrnSR orthologs are widely conserved in eukaryotic species except for excavates, whereas Imp13 orthologs are found only in unikonts (Supplementary Fig. S7). Thus, the functional divergence of TrnSR and Imp13 is likely to have emerged after the divergence to unikonts. However, many Imp13 cargo genes are found in unikonts and widely conserved in eukaryotic species. Moreover, we could not find any relevance between the phylogenetic profiles of the cargoes and the clustering results based on the mutant NTR-binding specificity. It might be possible that cargoes acquired after the divergence to unikonts were assigned to either TrnSR or Imp13 and also some of the cargoes already present before the divergence to unikonts were reassigned to Imp13. For this type of reassignment, both cargoes and NTRs changed gradually and coevolutionarily during the functional divergence. Consequently, some cargoes and NTRs may settle down to similar binding configurations. There were a few mixed pairs of TrnSR and Imp13 cargoes sharing sequence similarities (*p* value < 10^–15^), such as the RNA helicases DDX5 and DDX27 (Supplementary Table S3b). DDX5 and DDX27 are a homologous pair (34% sequence identity and *p* value = 9.9 × 10^–45^) and are widely conserved in eukaryotic species. Thus, DDX5 and DDX27 are likely to have been present before the functional divergence of TrnSR and Imp13. Those genes might have undergone alterations such as mutations on protein surfaces, gain/loss of domains or disorder regions by some constraints during the functional divergence to adapt to recognition by TrnSR or Imp13. Additionally, concave surfaces of NTRs might have changed following the alterations. Further coevolutionary analysis of the NTRs and the cargoes would be vital in uncovering how the functional divergence responsible for unique cargo recognition emerged.

## Methods

### Detection of amino acid sites involved in functional differentiation between TrnSR and Imp13

Seventy-eight and 72 amino acid sequences of metazoan TrnSR and Imp13 orthologs, respectively, were collected from the Inparanoid database^72^, and a multiple sequence alignment that included all of them was produced using MAFFT^73^. The amino acid composition at each alignment site was calculated within the two groups of orthologs, TrnSR and Imp13 (Fig. 1a and Supplementary Table 1). In this calculation, the weighting method for the residue count proposed by Gerstein et al.^74^ was used, and then the probability of each amino acid was estimated using Dirichlet mixture prior^75^. The difference between the TrnSR and Imp13 orthologs in the amino acid composition at each alignment site was evaluated by the modified KL value^60^, which is simply the Kullback–Leibler symmetric divergence defined as follows:

$$\mathop \sum \limits_{{i = 1}}^{{20}} p\left( i \right)log\frac{{p\left( i \right)}}{{q\left( i \right)}} + \mathop \sum \limits_{{i = 1}}^{{20}} q\left( i \right)log\frac{{q\left( i \right)}}{{p\left( i \right)}}$$

where *p*(*i*) and *q*(*i*) are the probability of amino acid *i* at an alignment site of the TrnSR and Imp13 orthologs, respectively. The KL values were used to predict the sites involved in the functional differentiation between TrnSR and Imp13. Sites at which more than half of the sequences had gaps were skipped.

### Plasmids and proteins

WT and mutant GST-mCherry-TrnSR and -Imp13 proteins were expressed from the pGEX-6p3 vector (GE Healthcare). Human TrnSR and Imp13 cDNAs carried in pGEX-6p3 were mutagenized using the KOD plus mutagenesis kit (Toyobo) and a PCR fragment encoding mCherry from the pmCherry-C1 plasmid (Clontech) was inserted between the GST and the TrnSR or Imp13 coding regions. The proteins were expressed in *Escherichia coli* BL21 and purified on GSH-Sepharose 4B (GE Healthcare) and a Mono Q column (GE Healthcare) as described previously^76^. GST-TrnSR proteins without mCherry-fusion used in the LSPR experiment were also purified similarly on GSH-Sepharose 4B.

GFP-cargo fusion proteins were also prepared as described previously^76^. cDNAs for the cargoes were amplified from a HeLa cDNA library (SuperScript, Life Technology) by PCR and inserted into the pQE80L vector (Qiagen) carrying the GFP gene. The protein sequences derived from the cloned DNA sequences are shown in Supplementary Table S2e. The proteins were expressed in *E. coli* BL21 and extracted by extraction buffer [50 mM Tris–HCl (pH 8.0, 4 °C), 500 mM NaCl, 1 mM EDTA, 10 mM 2-mercaptoethanol, 0.5 mM PMSF]. After dialysis against transport buffer (TB) [20 mM HEPES–KOH (pH 7.3), 110 mM KOAc, 2 mM MgOAc, 5 mM NaOAc, 0.5 mM EGTA], the extracts were cleared by a high-speed centrifuge. The GFP-cargo proteins in the extracts were analyzed and quantified by triplicate Western blotting with an anti-GFP monoclonal antibody (Roche, 1181446), a horseradish peroxidase-conjugated anti-mouse IgG antibody (BioRad, 170-6516), and Immobilon Western chemiluminescent HRP substrate (Millipore). Chemiluminescence images were acquired and analyzed by a Fusion Solo7S image analyzer (Vilber Lourmat). An extract containing intact GFP was used as the standard. The concentrations of GFP moiety and total protein in the extracts were normalized by adding an *E. coli* extract without GFP. Because the expression levels varied widely, we normalized the extracts into three conditions (Supplementary Table S2a and b). Extracts containing extensively degraded cargo were not analyzed. GFP-cargo proteins used only in the reproducibility analysis (Supplementary Fig. S4a–f, left panels) were prepared similarly but quantified by a fluorometer.

His_6_-tagged DNAJB1 and CBX1 proteins used in the LSPR experiment were expressed and extracted similarly and then purified on TALON metal-affinity resin (Clontech).

### Bead halo assay

The purified GST-mCherry-TrnSR or -Imp13 protein was mixed with GSH-Sepharose beads at a ratio of 5 mg protein to 1 mL (bed volume) beads in TB, and after incubation for 20 min with occasional mixing, the beads were washed three times with TB. The beads were then mixed with an excessive amount of the normalized extract in EHBN buffer^62^ (10 mM EDTA, 0.5% 1,6-hexanediol, 10 mg/mL BSA, and 125 mM NaCl). After incubation on ice for 30 min, the beads were observed by a confocal laser scanning microscope FX1200 (Olympus) with a 40 × objective UPlanSApo40X2 (NA 0.95). Images were acquired using FV10-ASW software and 473 and 559 nm laser lines for GFP and mCherry, respectively, with Kalman 3 filter mode. Twelve-bit images with pixel dimensions of 1024 × 1024 (317 × 317 µm) were used for quantitation with the Fiji package of ImageJ software^77^. ROIs for quantitation were set on the mCherry images. The outlines of the beads were selected using the Analyze Particle command with options: size = 5020–17,700 (diameter = 80–150 µm), circularity = 0.80–1.00, and "exclude on edges". Ring-shaped regions with a width of 5 pixels along the inside of the outlines were fixed as ROIs, and larger ring-shaped regions with the same width but 5 pixels away outside the outlines were established as BG regions (Fig. 3 and Supplementary Fig. S2). The same ROIs and BG regions were applied to the corresponding GFP images. In the final assay, ~ 55%, ~ 40%, ~ 4.6%, and ~ 0.15% of the images included one, two, three, and four beads as the ROIs, respectively. The mean intensities of these regions in an image were quantified, and the value of GFP/mCherry = (ROI_GFP_-BG_GFP_)/(ROI_mCherry_-BG_mCherry_) was used as the index for NTR–cargo binding. Three images for one NTR–cargo pair were quantified and averaged, and then the GFP/mCherry values of NTR mutants were normalized by dividing by that value of WT NTR binding to the same cargo.

### Statistical analysis of the results of bead halo assay

The Mann–Whitney U test was employed to determine the statistical significance of the differences in GFP/mCherry values between WT and each mutant NTR. In the reproducibility analysis (Supplementary Fig. S4a–f, left panels), all the GFP/mCherry values in triplicate were pooled and tested. In the final assay (Supplementary Table S2a and b), three values of each WT and mutant NTR were tested, and the possible minimum *p* value was 0.05.

### Localized surface plasmon resonance

LSPR experiments were performed on an OpenSPR instrument (Nicoya) using NTA sensor tips (SEN AU NTA). TB containing 0.05% Tween-20 and excluding EGTA flowed at 20 µL/min as the running buffer, and proteins were injected for 217 s at 600-s intervals into the flow cell. After the sensor was charged with Ni^2+^ ions, 50 µg/mL His_6_-DNAJB1 was injected into channel 2, His_6_-CBX1, which does not bind to TrnSR (Supplementary Table S2a), was injected into channel 1 as a reference, and then varying concentrations of GST-TrnSR (see Supplementary Fig. S5a and b) were injected into channels 1 and 2 to observe the association. Dissociation in the buffer flow was observed continuously. The sensor tip was regenerated by 200 mM imidazole and 10 mM glycine–HCl (pH 1.5) and reused no more than three times. The resonance intensity of channel 2 was corrected by subtracting that of channel 1, and the kinetic curves were fitted to the one-to-one model using TraceDrawer 1.8 (Ridgeview Instruments) to derive k_on_, k_off_, and K_D_.

### Hierarchical clustering of the cargoes based on the difference in binding to the NTR mutants

The cargoes were hierarchically clustered using Ward's method as implemented in the software R (version 3.5.1)^78^. Based on the experimentally estimated deviation of the BHA values (Fig. 4a–d), the normalized GFP/mCherry ratios (Supplementary Table S2a and b) were converted into three discrete values: GFP/mCherry ratios ≤ 0.2 into 0, > 0.2 and ≤ 0.6 into 0.5, and > 0.6 into 1. The Euclidean distances between the cargoes calculated from these discrete values were used as the metric.

### Sequence and structural similarity search and disorder prediction

Sequence similarities among the cargo proteins were computed using SSEARCH 36.3.8d^79^, and sequences with an E-value < 10^–9^ were judged as similar. Disordered regions were predicted using DISOPRED 3.1^80^. For 3D-structural comparison, structures were aligned using MICAN^70^, and the similarity was evaluated by the TM-score of MICAN. Structures with TM-scores > 0.6 were judged as highly similar and > 0.5 as similar. For protein regions whose structures are unknown, templates for structural modeling were selected based on HHpred^81^ alignment, and the structures were modeled using Homology Model application in Molecular Operating Environment (MOE 2016.08; Chemical Computing Group ULC, 1010 Sherbooke St. West, Suite #910, Montreal, QC, Canada, H3A 2R7).

### Evolutionary profiles of TrnSR, Imp13, and their cargoes

Orthologous sequences of TrnSR and Imp13 were searched using HMMER-2.2.0 for glocal alignment^82^. For the glocal profile, an HMM was generated from a multiple sequence alignment produced using MAFFT^73^ with manually curated orthologous sequences. Sequence search-based ortholog identification of the cargo proteins was conducted automatically using OrthoMCL 2.0.9^83^ with our proteome data sets in a previous study^84^.

### RSY-rich segment search

The sequences of TrnSR cargoes were scanned with sliding windows of 15–30 amino acids in length, and then the composition of R, S, and Y (RSY_comp_), dipeptide composition of RS, SR, SY, and YS (dipepRSY_comp_), and SY and YS (dipepSY_comp_) were calculated to search for RSY-rich segments^25^. Fragments with RSY_comp_ > 0.3, dipepRSY_comp_ > 0.2, and dipepSY_comp_ > 0 were defined as RSY-rich segments.

## Supplementary Information

Supplementary Information 1.

Supplementary Information 2.

Supplementary Information 3.

Supplementary Information 4.
