# Supplementary material for: Distinct mutations in importin-β family nucleocytoplasmic transport receptors transportin-SR and importin-13 affect specific cargo binding
Source: Sci Rep. 2021 Aug 2;11:15649. doi: 10.1038/s41598-021-94948-1 (PMC8329185; doi:10.1038/s41598-021-94948-1)
Supplement: Supplementary file 1 — Supplementary Information 1. [file 41598_2021_94948_MOESM1_ESM.pdf]

Distinct mutations in importin-β family nucleocytoplasmic transport receptors transportin-SR and importin-13 affect specific cargo binding

Makoto Kimura, Kenichiro Imai, Yuriko Morinaka, Yoshiko Hosono-Sakuma, Paul Horton, and Naoko Imamoto

Supplementary figures

Fig. S1: Western blotting of bacterially expressed GFP-cargoes. (2 sheets)

Legend for Fig. S1.

Fig. S2: Examples of ROI and BG settings.

Legend for Fig. S2.

Fig. S3: Threshold for negative binding.

Legend for Fig. S3.

Fig. S4: Reproducibility of bead halo assay.

Legend for Fig. S4.

Fig. S5: LSPR analysis of TrnSR–DNAJB1 interaction.

Legend for Fig. S5.

Fig. S6: Binding of the cargoes to the WT NTRs in the presence or absence of RanGTP. (4 sheets)

Legend for Fig. S6.

Fig. S7: Phylogenetic profiles of the orthologs of TrnSR, Imp13, and their cargoes.

Legend for Fig. S7.

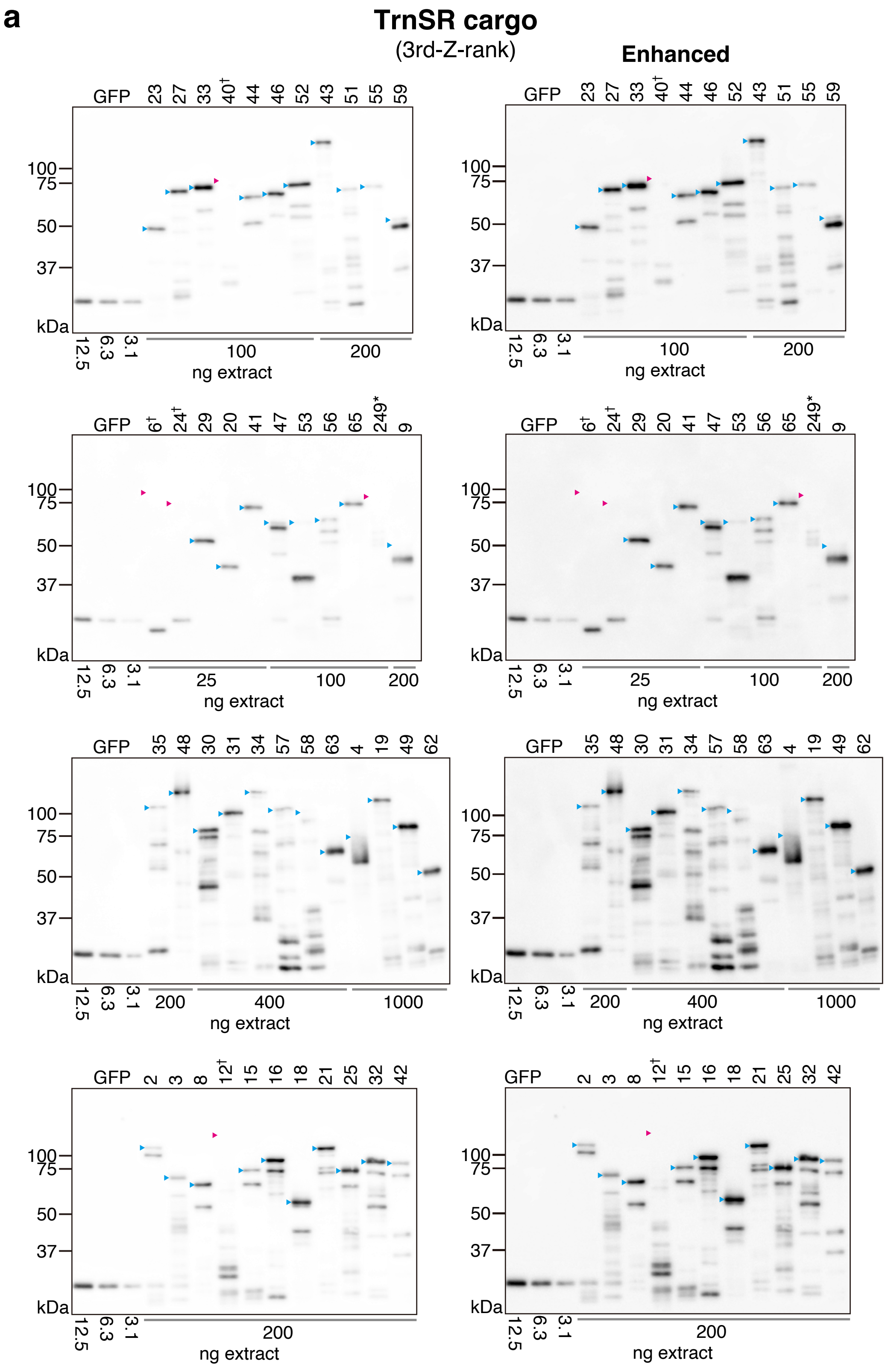

Supplementary Fig. S1 (1/2)

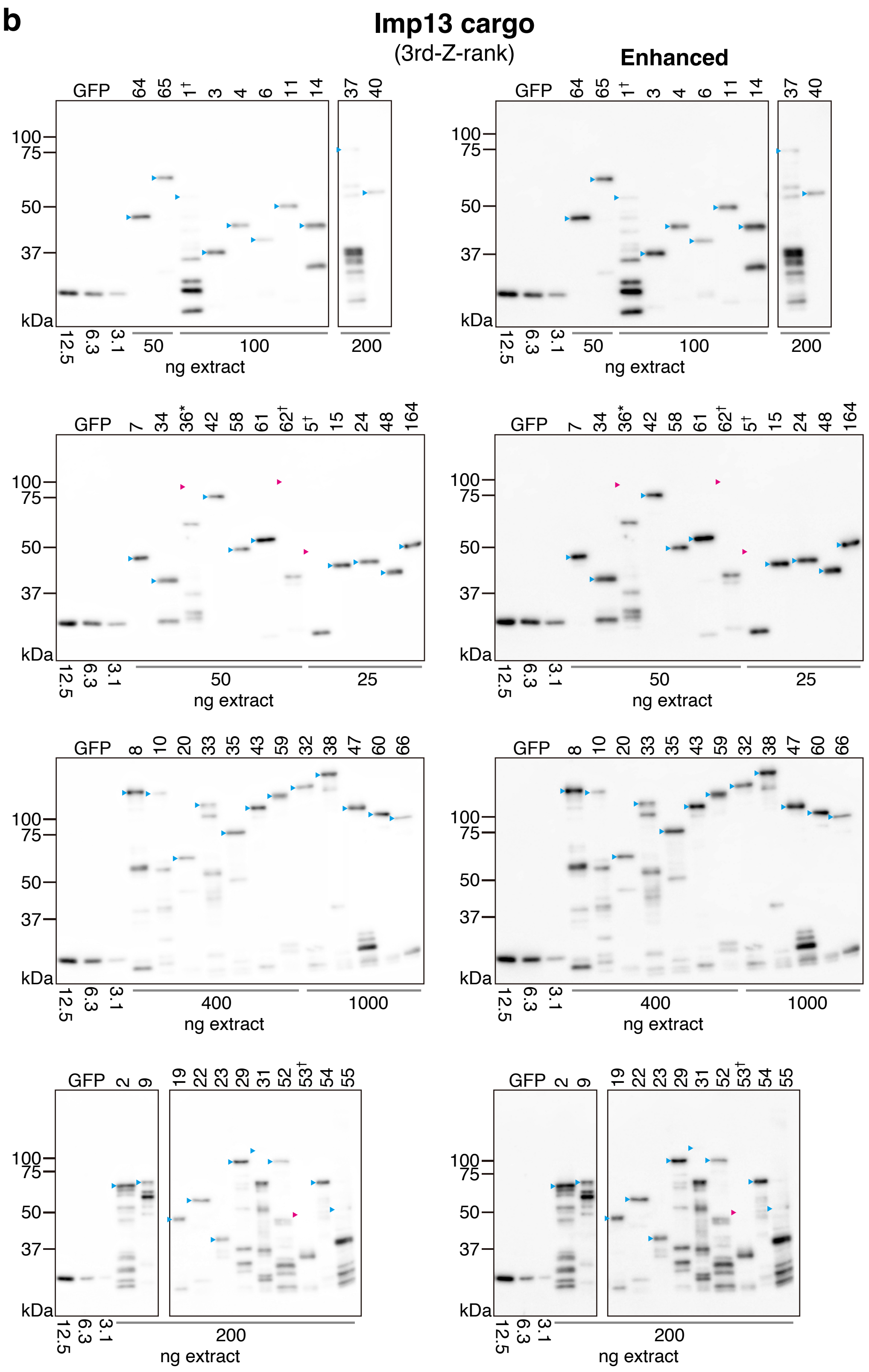

Supplementary Fig. S1 (2/2)

Supplementary Figure S1. Western blotting of bacterially expressed GFP-cargoes.

GFP-cargo proteins in the bacterial extracts were analyzed by Western blotting using an anti-GFP antibody.

(a) TrnSR cargo.

(b) Imp13 cargo.

(Left panels) Unprocessed images.

(Right panels) Enhanced images (range: 0–80%, γ value: 0.7).

Chemiluminescence images were acquired and analyzed by an image analyzer. The cargo proteins are specified by their 3rd-Z-rank orders (see Supplementary Table S2a and b) at the top, and the total protein amounts of the loaded extracts are shown at the bottom. An extract containing intact GFP was used as the quantitation standard. The band with the slowest mobility within the expected molecular weight range is presumed as the full-length protein and indicated by a cyan arrowhead. In lanes where no such band is present, the expected position for the full-length protein is indicated by a magenta arrowhead. Bands which migrated much faster than expected were regarded as degradation products. Daggers indicate extensively degraded proteins that were not analyzed by BHA. Degraded proteins indicated by asterisks were analyzed by BHA, because the major fragments retained more than half portion. Unrelated lanes were cropped. One representative image of three is shown. The TrnSR 36th cargo is the same protein as the Imp13 38th cargo.

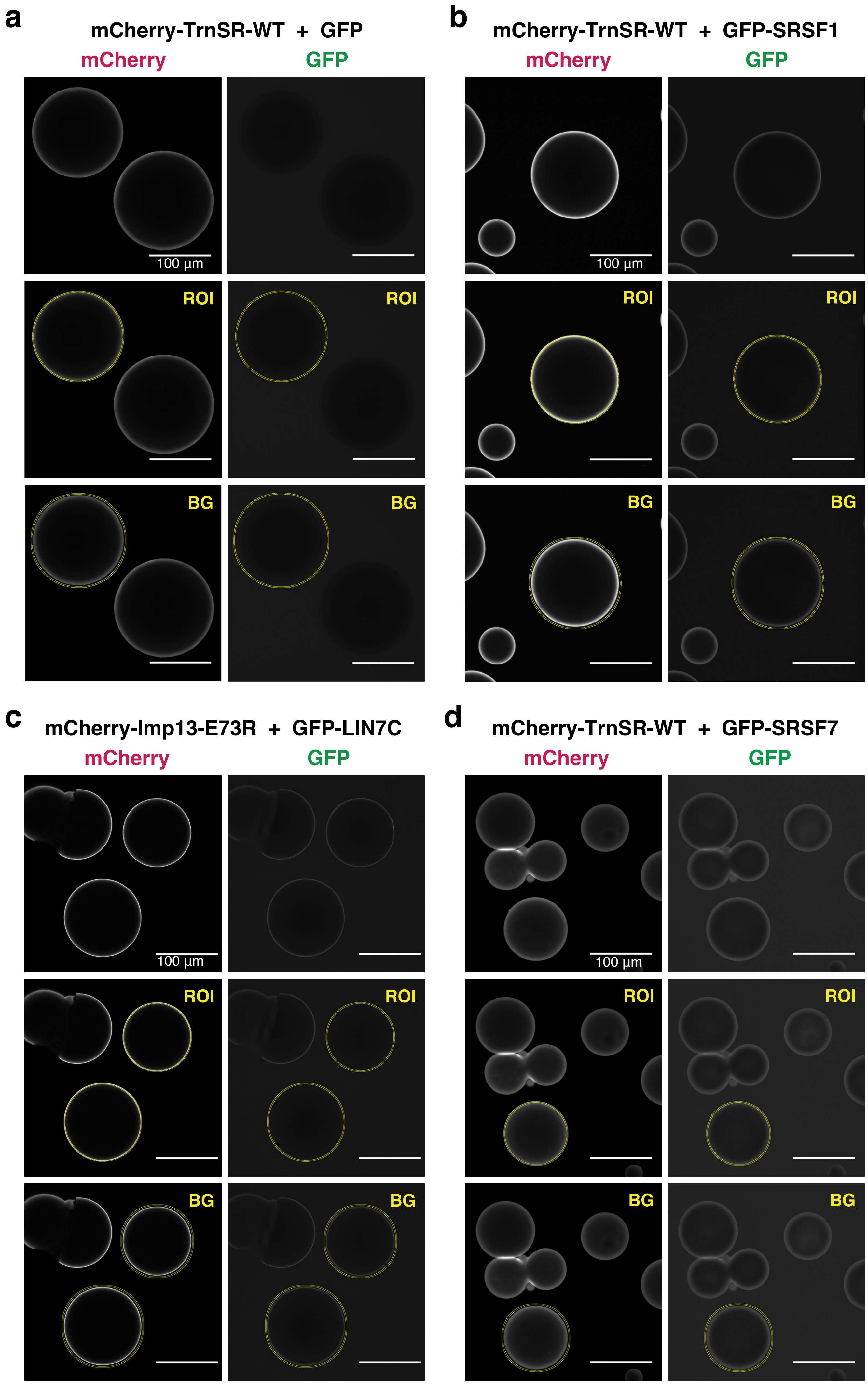

Supplementary Fig. S2

Supplementary Figure S2. Examples of ROI and BG settings.

Images of GFP and mCherry fluorescence that illustrate typical ROI settings are collected. ROIs and BGs are indicated on the same mCherry or GFP fluorescent images by double yellow lines.

(a) Only beads 80–150 μm in diameter are selected. The same images are shown in Fig. 3c and d.

(b) Only beads not on the edge of the image are selected. The same images are displayed in Fig. 3g and h.

(c) Only unbroken beads (circularity 0.8–1.0) are selected, and two or more beads in one image can be selected if they satisfy the conditions. ZNF281 was not analyzed further because it degraded extensively (Supplementary Table S2a).

(d) Only beads separated enough from other beads to set the BG not overlapping with the other beads are selected.

The NTR and cargo used are indicated on the top. Scale bar: 100 μm.

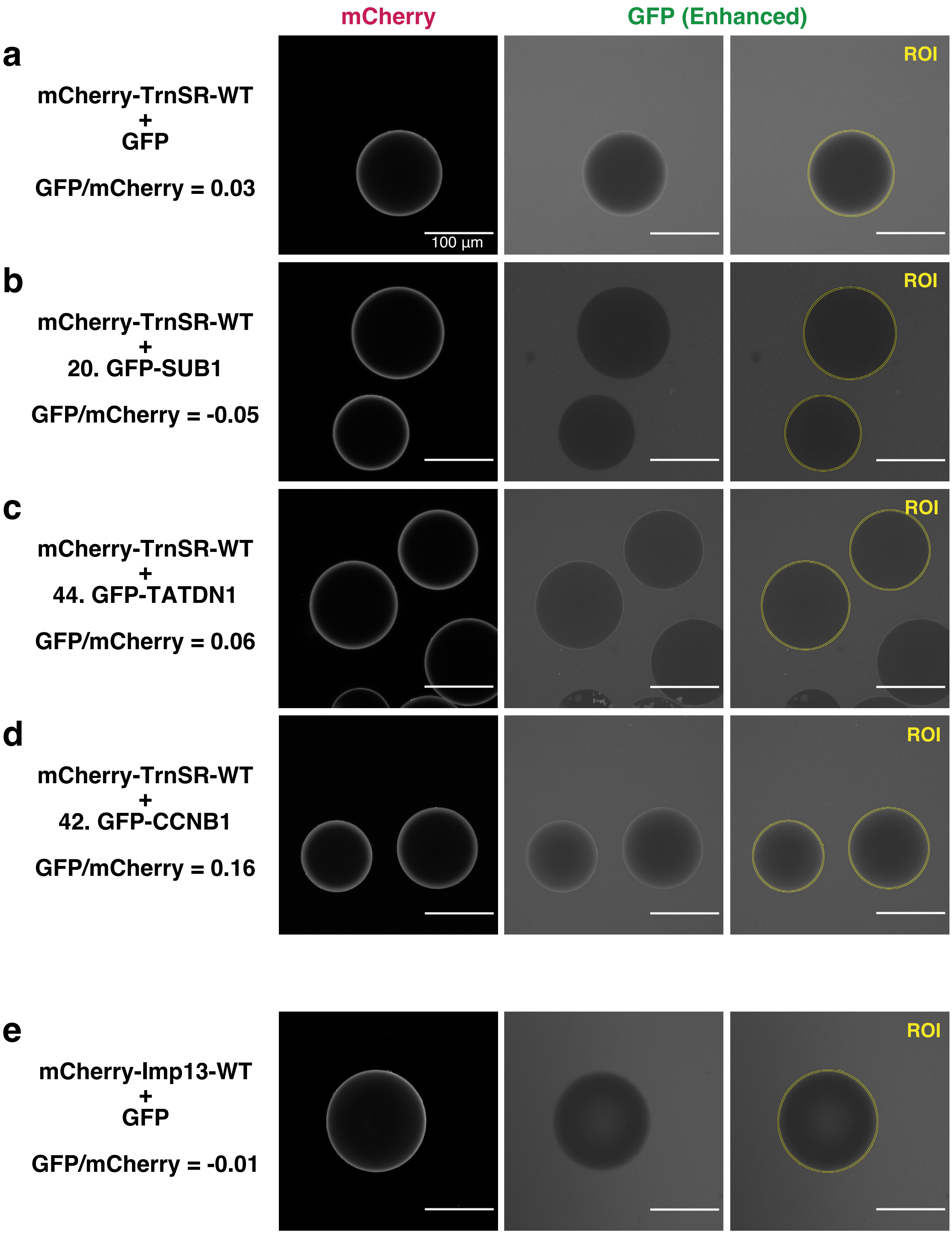

Supplementary Fig. S3

Supplementary Figure S3. Threshold for negative binding.

Images of BHAs that illustrate negative or faint binding are collected. The GST-mCherry-NTR and GFP-cargo (number: 3rd-Z-rank) used are denoted with the GFP/mCherry ratio (not normalized) calculated from three images. Yellow double lines signify ROIs on the GFP images.

(a) The negative control, GFP, shows a faintly positive GFP/mCherry ratio for TrnSR binding.

(b) A candidate cargo, SUB1, exhibits an obviously negative ratio.

(c) TATDN1 exhibits a ratio near that of GFP.

(d) CCNB1 displays an obviously positive ratio. The same images are shown in Supplementary Fig. S6.

(e) GFP exhibits a negative ratio for Imp13 binding, and thus, GFP has a higher affinity for TrnSR than for Imp13.

We set the GFP/mCherry ratio thresholds for negative binding at 0.1 for TrnSR and 0.05 for Imp13 to avoid unreliable quantitation at lower range. The GFP images are enhanced equivalently. Scale bar: 100 μm.

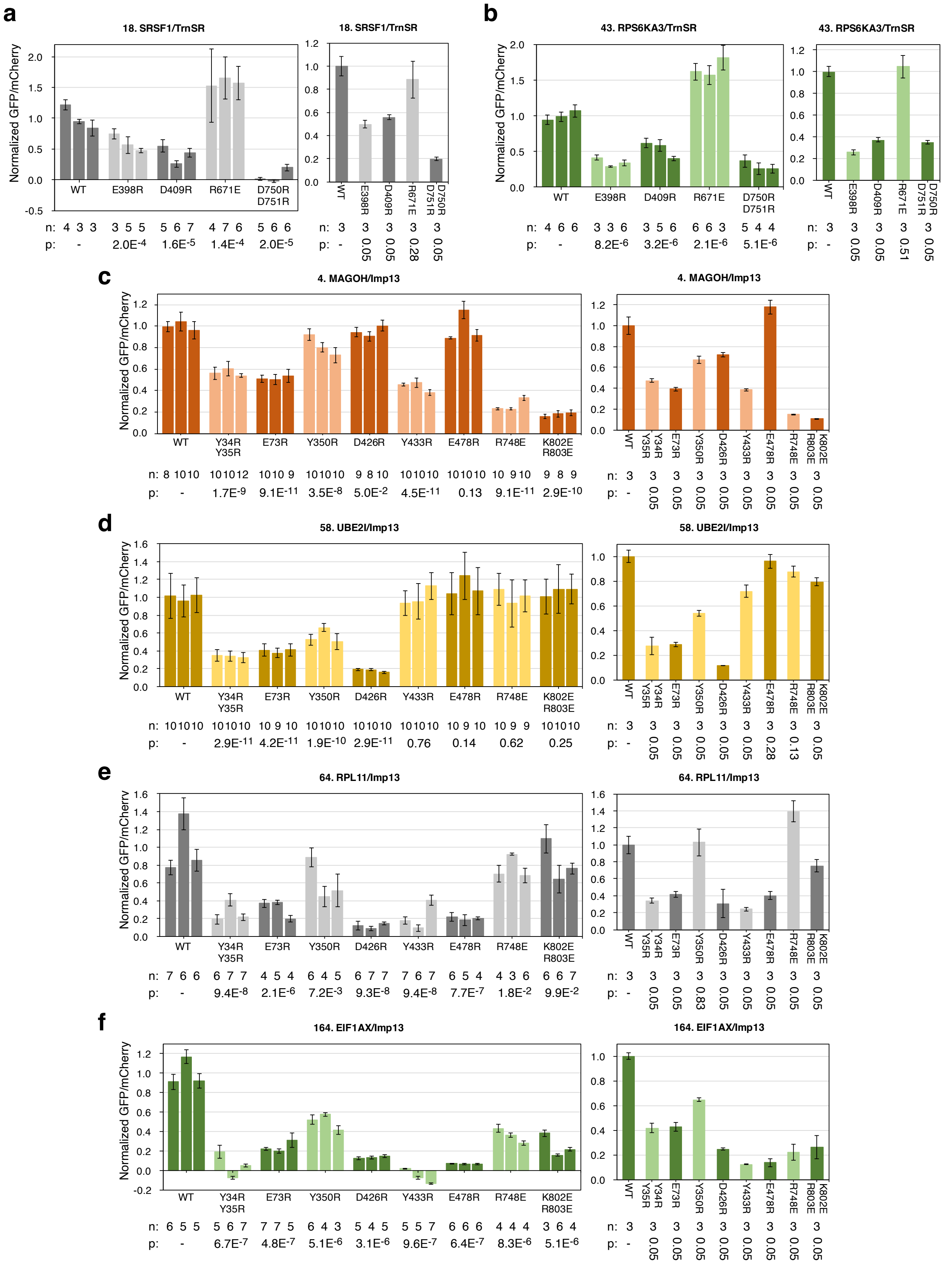

Supplementary Fig. S4

Supplementary Figure S4. Reproducibility of bead halo assay.

(a-f, left panels) Each BHA was triplicated using the same GST-mCherry-NTR protein and extract containing GFP-cargo. The extracts were prepared independently of those used in the final assays, and used only in these reproducibility analyses. A fluorometer quantified GFP-proteins in the extracts. The GFP/mCherry ratios were normalized to the mean value of the WT triplicate. Two TrnSR (a and b) and four Imp13 (c-f) cargoes were analyzed.

(right panels) Results of the final assay (Supplementary Table S2a and b). GFP-proteins were quantified by triplicate Western blotting. The GFP/mCherry ratios were normalized to that of WT. The NTR and cargo analyzed are indicated at the top, and the NTR mutations are at the bottom. Error bar, S.D.; n, number of analyzed images; p, p-value of Mann–Whitney U test. In the left panels, all the GFP/mCherry values in triplicate were pooled, and the significance of the difference between the WT and each mutant was evaluated. In the right panels, the minimum p-value is 0.05 because n=3 in all the assays. E, ×10.

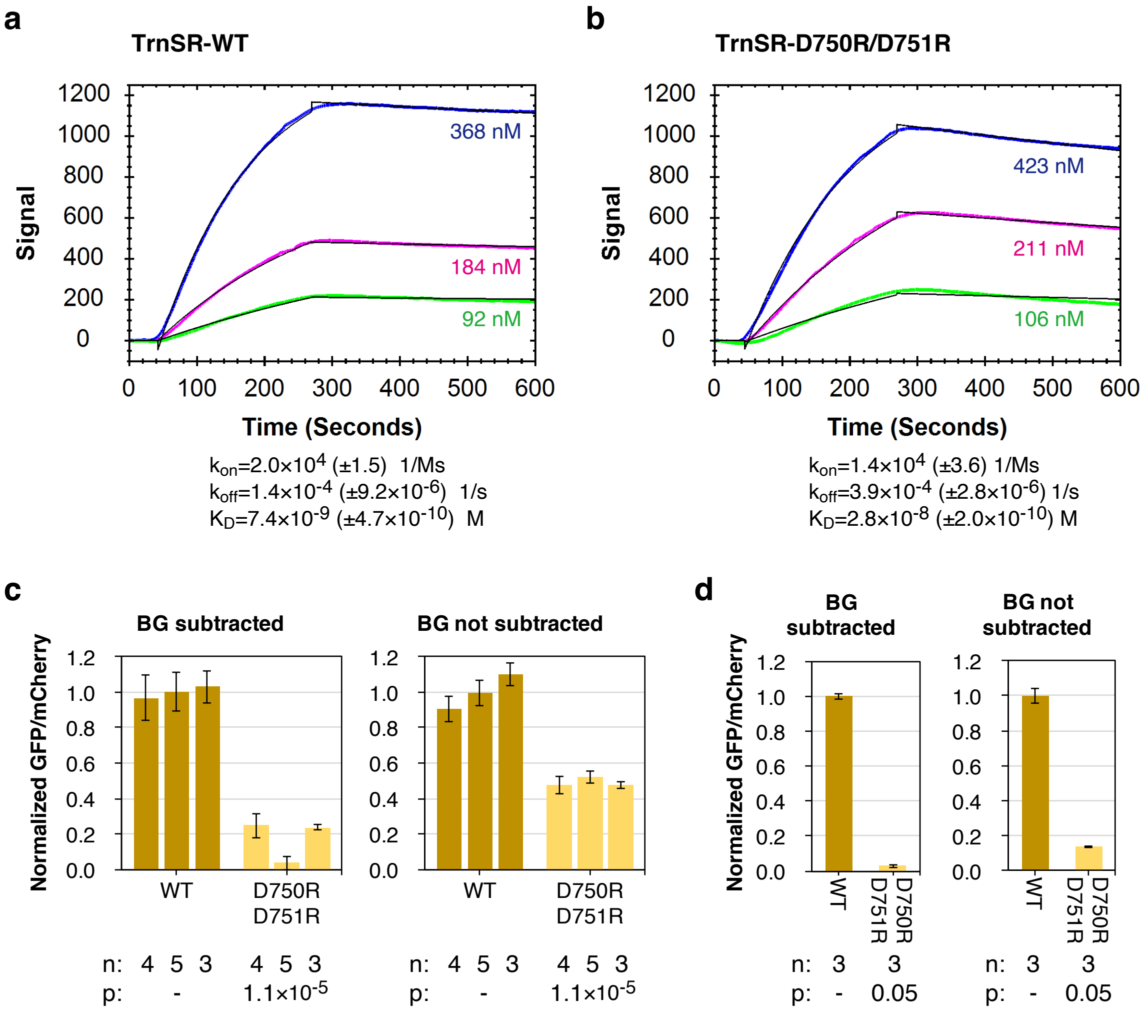

Supplementary Fig. S5

Supplementary Figure S5. LSPR analysis of TrnSR–DNAJB1 interaction.

(a, b) LSPR analyzed kinetic interaction between the TrnSR cargo DNAJB1 and TrnSR-WT (a) or TrnSR-D750R/D751R (b). His_6-DNAJB1 was fixed on Ni^{2+}-charged NTA in channel 2 of the flow cell, whereas His_6-CBX1 was fixed in channel 1 as the negative reference. The indicated concentrations of GST-TrnSR-WT or -D750R/D751R flowed into channels 1 and 2 for 217 sec, and then the buffer flowed continuously. Signal intensity is presented by resonance units, and the signal intensity curve of channel 2 corrected by that of channel 1 is presented for each concentration of TrnSR. Curves fitted to the one-to-one model are drawn by thin black lines. The k_{on}, k_{off}, and K_D derived from the three curves are shown at the bottom. In the parentheses are the fitting errors.

(c) Binding of DNAJB1 to TrnSR-WT or -D750R/D751R was analyzed by BHA trice, similar to the reproducibility analysis shown in Supplementary Fig. S4.

(d) Binding of DNAJB1 to TrnSR-WT or -D750R/D751R in the final assay (Supplementary Table S2a).

In (c) and (d), the BG-subtracted GFP values were used in the left panels, whereas the BG values were not subtracted from the GFP values in the right panels.

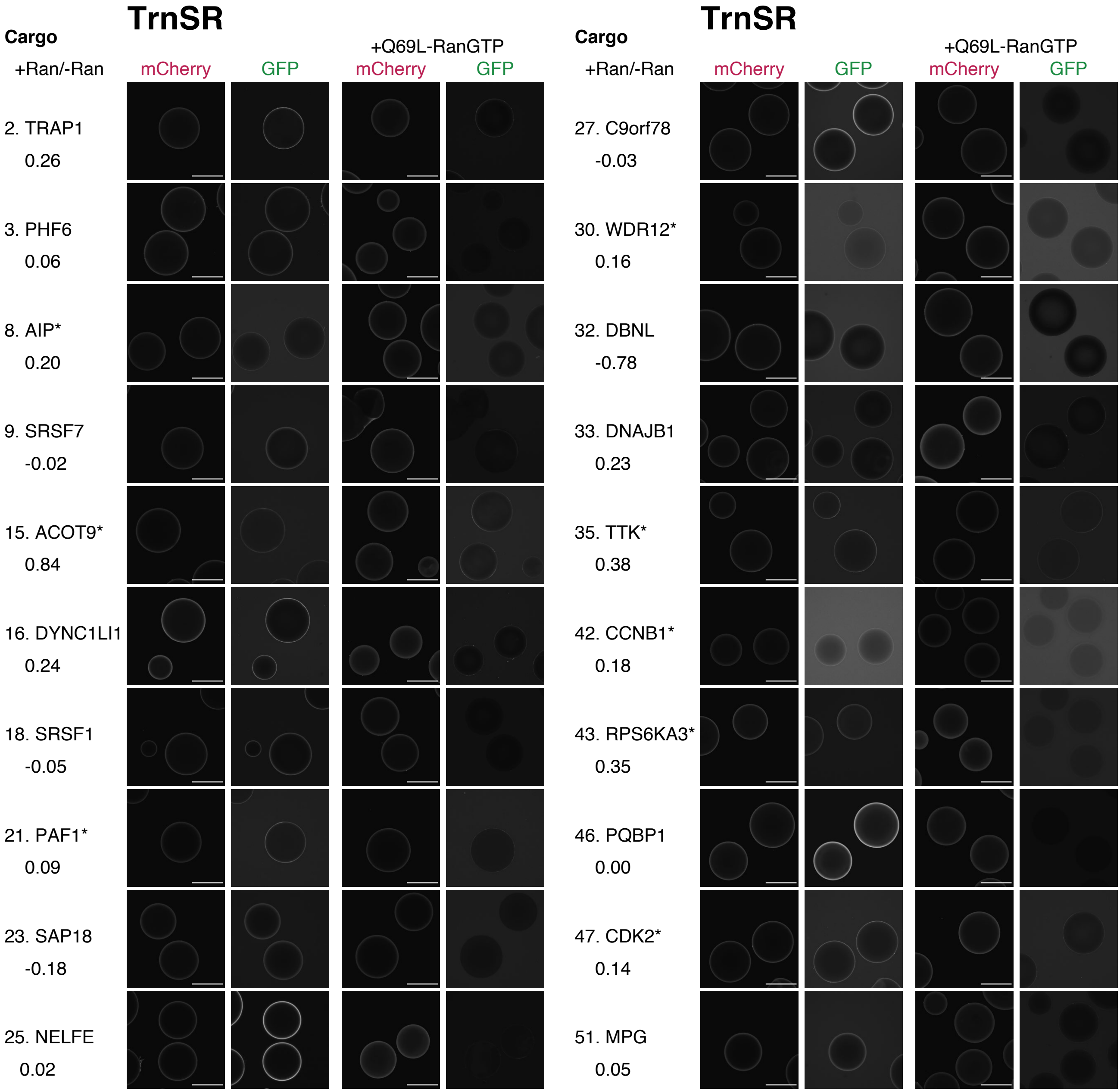

Supplementary Fig. S6 (1/4)

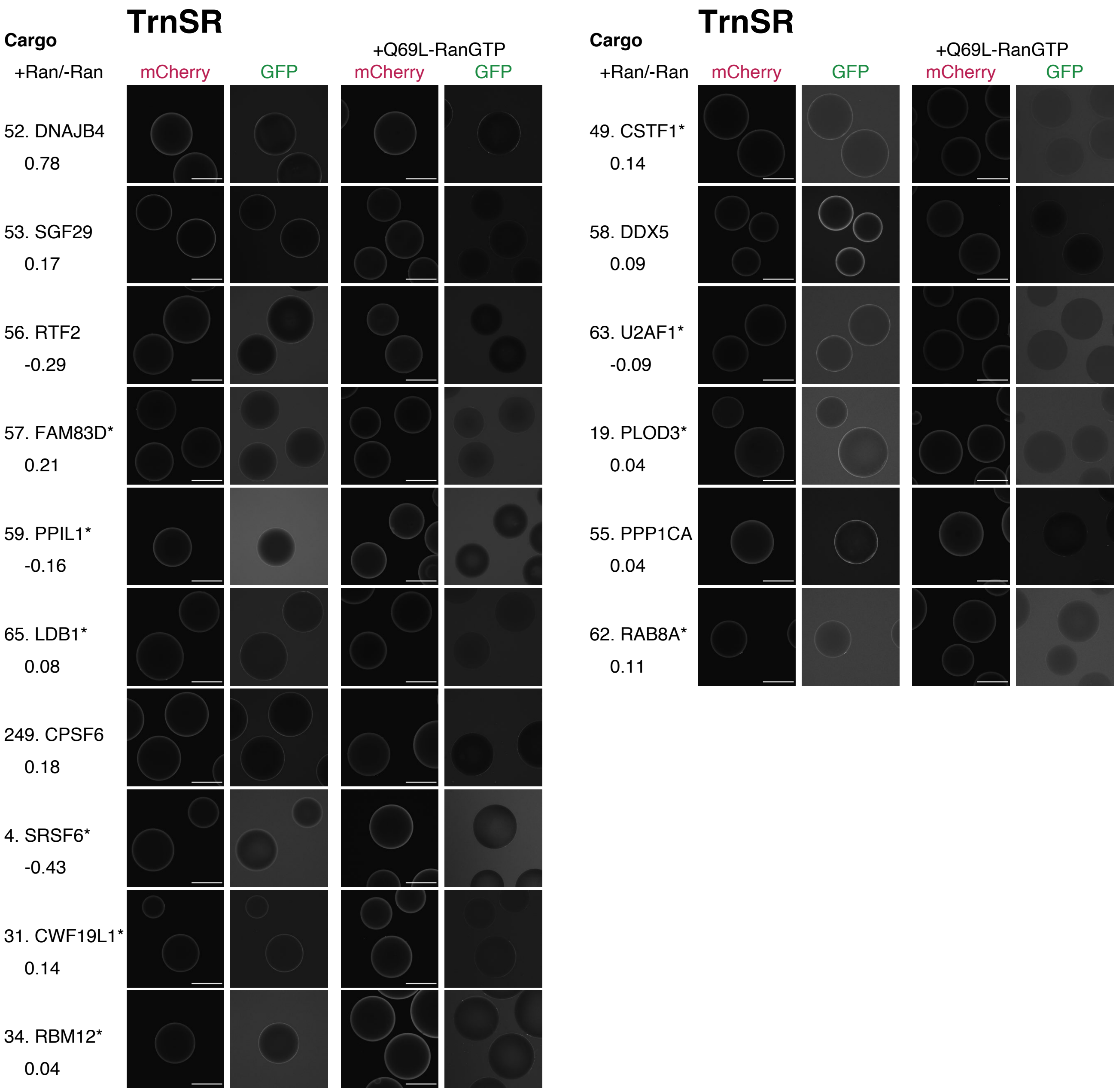

Supplementary Fig. S6 (2/4)

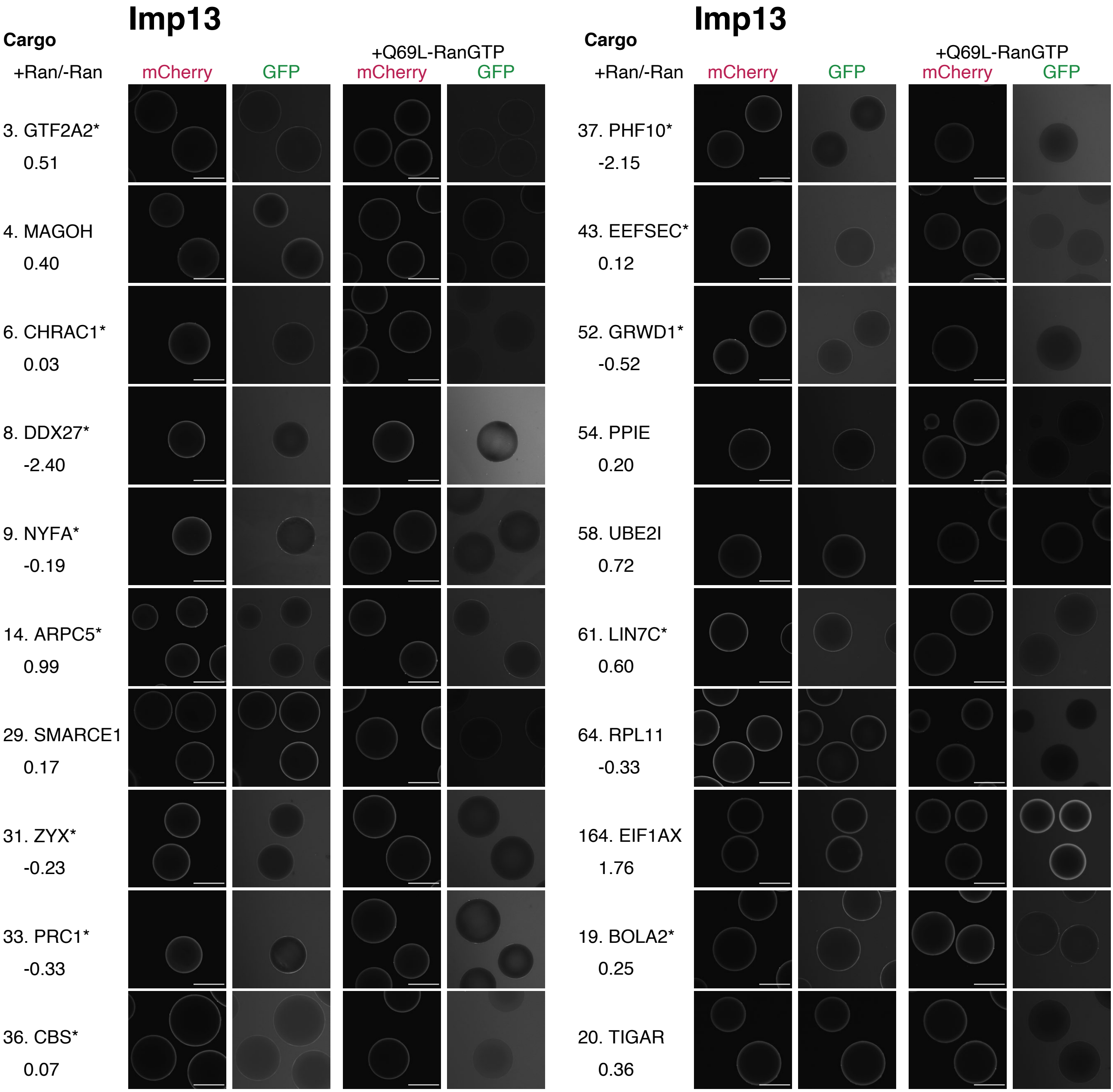

Supplementary Fig. S6 (3/4)

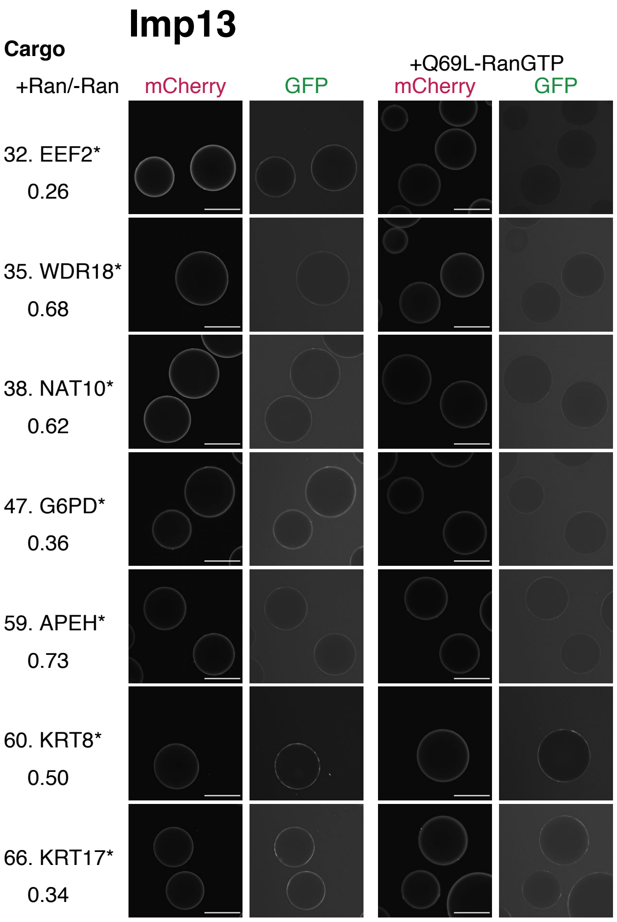

Supplementary Fig. S6 (4/4)

Supplementary Figure S6. Binding of the cargoes to the WT NTRs in the presence or absence of RanGTP.

Binding of the 3rd-Z-4% cargoes to TrnSR-WT or Imp13-WT was analyzed by BHA in the presence or absence of Q69L-RanGTP, which is a GTP-fixed mutant of Ran. RanGTP inhibits the binding of import cargoes to NTRs but promotes the binding of export cargoes. Bead halo images of the cargoes positively bound to the NTR are presented in the same order in Supplementary Table S2a and b (number: 3rd-Z-rank). GFP/mCherry ratios in the presence and absence of Q69L-RanGTP were measured from the images presented here, and their ratio (+RanGTP/-RanGTP) is shown. The asterisk indicates that the GFP images of ±Q69L-RanGTP are enhanced equivalently. Scale bar: 100 μm.

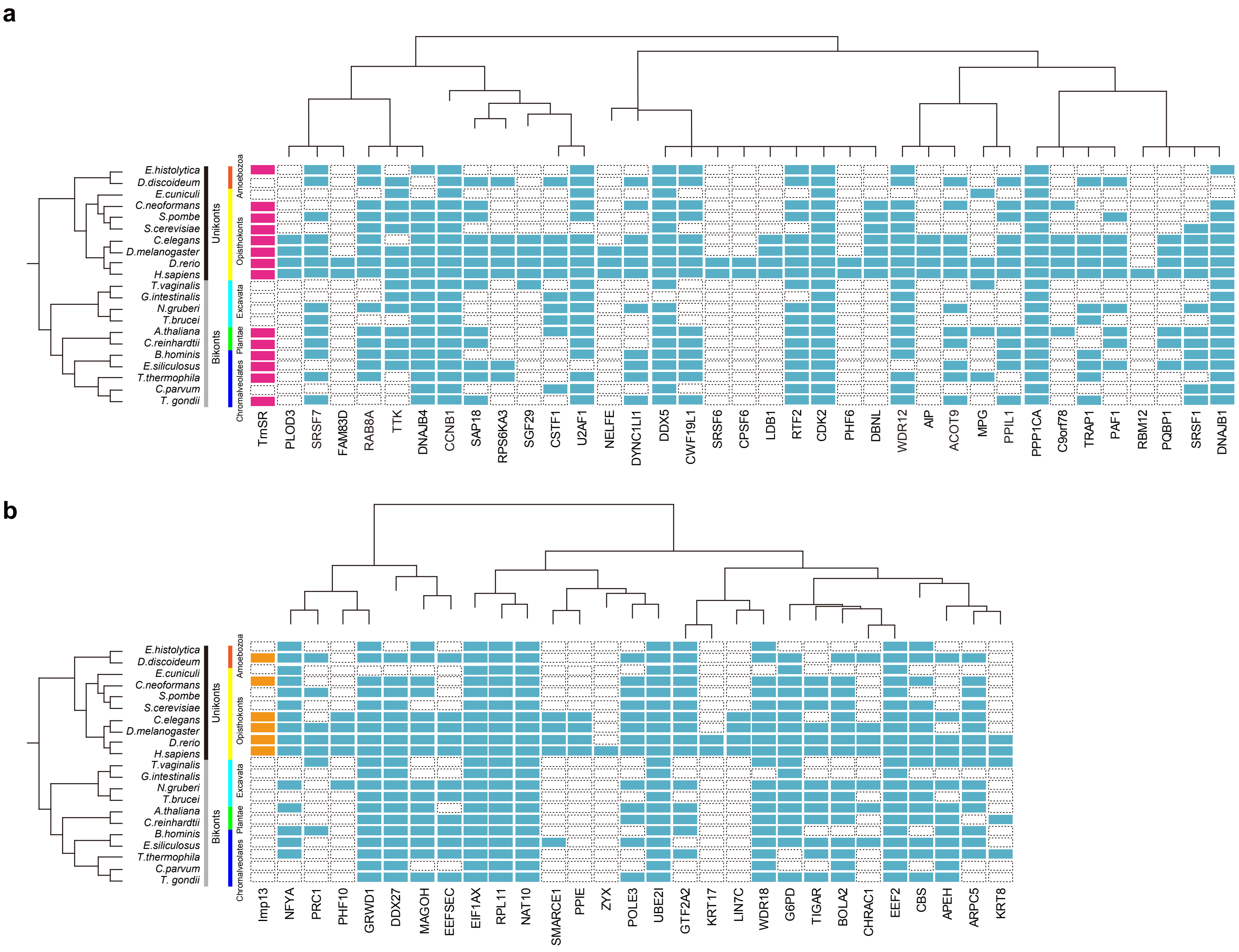

Supplementary Fig. S7

Supplementary Figure S7. Phylogenetic profiles of the orthologs of TrnSR, Imp13, and their cargoes.

(a) Beside the phylogenetic dendrogram of eukaryotes (left), species with orthologs of TrnSR and its cargoes are denoted by colored boxes. Taxa of the species are presented to the right of the scientific names. An upper dendrogram of the cargo clustering was generated based on the mutant TrnSR-binding profile (Fig. 7a).

(b) Imp13 and its cargoes are shown as in (a).
